# A systematic evaluation of five different image-derived input functions for the clinical implementation of ^18^F-NaF bone PET/CT in patients with chronic kidney disease–mineral and bone disorder

**DOI:** 10.3389/fnume.2023.1235800

**Published:** 2023-07-28

**Authors:** Jørn Theil, Marie Houmaa Vrist, Jesper Nørgaard Bech, Claire Anne Fynbo

**Affiliations:** ^1^ Department of Nuclear Medicine, Gødstrup Hospital, Herning, Denmark; ^2^ Department of Clinical Medicine, Aarhus University, Aarhus, Denmark; ^3^ University Clinic in Nephrology and Hypertension, Department of Medicine, Gødstrup Hospital and Aarhus University, Herning, Denmark

**Keywords:** [^18^F] sodium fluoride, PET imaging, bone metabolism, kinetic analysis, modeling, input function, comparative analysis, CKD-MBD

## Abstract

**Introduction:**

The aim of this study was to investigate the impact of the use of varying input parameters on resulting bone plasma clearance (*K_i_*) and other kinetic modelling parameters in a group of patients with chronic kidney disease–mineral and bone disorder (CKD-MBD).

**Methods:**

Raw PET/CT data and blood data were systematically analyzed using five different VOIs for the input functions in the left ventricle and in the thoracic aorta. Standardized VOIs were placed in four thoracic vertebrae and the results pooled and averaged. The basic image-derived input functions (IDIFs) were corrected for partial volume effect and spill-over and modified by substitution of the terminal image exponential with the corresponding plasma-exponentials derived from blood samples. *K_i_* was then calculated using both a non-linear regression (NLR) analysis and a graphical Patlak analysis and compared.

**Results:**

Our original results were reproducible with an inter-observer difference of approximately 6%. The correction factors varied with the VOI volumes from 0.73 ± 0.17 for the largest LV-VOI (48.7 ± 25.3 cm^3^) to 0.99 ± 0.10 for the AO-VOI (3.4 ± 1.2 cm^3^). The mean NLR-*K_i_* results varied between 0.0378 ± 0.0112 and 0.0432 ± 0.0095 ml/min ml^−1^ with a fixed vB and 0.0408 ± 0.0111 and 0.045 ± 0.0102 ml/min ml^−1^ with a free-fitted vB. The corresponding Patl-*K_i_*-results varied between 0.0302 ± 0.0071 and 0.0325 ± 0.0070 ml/min ml^−1^, having lesser differences and variances. The input functions with least variance and mean differences compared with NLR results were derived from the left ventricle with a VOI volume of 19.2 ± 11.3 cm^3^ corrected for PVE and Bg with a mean *K_i_*-difference: 0.0097 ± 0.0370 ml/min ml^−1^ and 95% confidence limits (−0.023 to 0.004).

**Conclusions:**

Our results indicated that a VOI with a volume of approximately 20 cm^3^ with a correction factor of 0.83 ± 0.13 results in Patlak results with the least variance and difference compared with the NLR results. The use of free-fitted vB in the NLR analysis showed the most robust results in all input series. The Patlak results were in comparison generally lower than the NLR results (−17.3% to −23.4%) but very robust across the various input series and with results comparable to previously published data and are therefore recommended for future analysis.

## 1. Introduction

The practical implementation of correct and accurate quantification of dynamic positron emission and computed tomographic (PET/CT) tracer kinetic studies of metabolism is non-trivial, the success of which is dependent on the choice of many analysis-input parameters, both scanner and protocol related, all of which need to be understood and optimized when locally implementing a new examination method for either research or clinical purposes.

Historically, the use of dynamic PET/CT tracer kinetic studies has been restricted primarily to research purposes at larger, specialized university departments with many years of experience in the necessary local implementation and development of analysis protocols and software, with the clinical use of dynamic PET/CT techniques being restricted to a few commercially developed functional imaging packages for a limited range of organs, e.g., ^18^F-FDG-PET/CT (fluorodeoxyglucose) and ^82^Rb-PET/CT (^82^Rubidium) for myocardial perfusion examinations. Currently, with the more widespread availability of PET/CT scanner functional options (list-mode data acquisition, vendor supplied re-binning reconstruction software, simplified kinetic analysis software tools), the use of a kinetic analysis of dynamic PET/CT acquisitions for other organs, with various radiopharmaceutical tracers, is becoming an attractive and attainable possibility in the more general clinical setting. However, before deviating from the use of the commercially available application packages, a number of technical questions need to be addressed.

Within the field of studying bone metabolism, a recent paper by Puri et al. (1) answered in detail some of the questions we needed answers to when we, more than 5 years ago, implemented dynamic tracer kinetic studies of metabolism using ^18^F-NaF-PET/CT in a group of patients with chronic kidney disease–mineral bone disorder (CKD-MBD). The goals of our original study (2) were to implement methods for determining bone plasma clearance (*K_i_*) in this patient population, based on the methods as previously applied to a population of patients with osteoporosis (3–5) and ultimately to derive a standardized input curve (“semi-population input function,” see Section 2.1.4) for future routine use in a site-specific analysis of patients with CKD-MBD (6).

To achieve these original goals, our volume-of-interest (VOI) definition used a contour in the range of 45%–65% of the maximum value within a box limiting the left ventricle of the heart (LV). This resulted in a VOI filling most of the LV, which was separated from the background VOI in the myocardial wall by at least 2 voxels (≥6.4 mm). The VOIs were subject to partial volume effect (PVE) and spill-over of background activity (Bg) to and from the surrounding myocardial wall and it was necessary to correct for PVE and Bg as described by Cook et al. (3) through the use of a measured recovery coefficient (RC_ß_). The mean subject-specific measured RC_β_ was 0.69 ± 0.15 (2), which seemed a little low for a modern PET/CT scanner when using ^18^F-NaF as a tracer.

In addition, our original work used a fixed blood-volume fraction parameter (vB) of 0.05 instead of a free-fitted vB in the non-linear regression analysis (NLR). Our implemented method of substituting the final image exponentials of the input curves with exponentials derived from plasma samples using logarithmic transformation differed from that described by Frost et al. (4) and Blake et al. (6).

In the review process for publication of this original study, this selection of parameters and method implementation, which were based on the available literature at the time (3, 6), was questioned, raising discussion and debate regarding the following: (1) the choice of VOI definition and size, (2) the best/most correct way to correct for PVE and spill-over from the background, and (3) the most correct value for the fractional blood volume parameter (vB).

As these issues were considered limitations in our original study, this work presents an attempt to improve and clarify the following points: (1) the effects of VOI definition, size, and correction factor on the input functions and the dynamic results; (2) the difference caused by the use of fixed vB vs. free-fitted vB; (3) whether the results obtained with our “logarithmic multiplicative method” are different from the results obtained with the original “exponential additive method” as described by Blake et al. (6); (4) whether the inclusion of an additional blood sample at 90 mpi (minutes post injection) at the end of the whole-body scan results in a better fit between image and blood data; and (5) whether a semi-population function (SP-function) derived from the optimized analysis of CKD-MBD patients differs from the corresponding SP-function derived from patients with osteoporosis.

The latter point is important, in order to indicate the necessity, or not, of requiring separate SP-functions for individual disease populations, as differences in bone turn-over may affect the shape, and thus the area under the curve (AUC) of the plasma curve (7).

## 2. Methods

### 2.1. Patients

Raw data from 12 patients with CKD-MBD enrolled in our previous study of method implementation (2) were reanalyzed for reproducibility. For the optimized analyses in this study, one obese patient was excluded due to extremely poor counting statistics resulting in outlying data in all series with small VOI volumes ≤1 ml.

### 2.2. Blood samples

In the original study, 5-ml venous blood samples were collected at −5, 30, 40, 50, 60, and 90 min after injection and prepared for well counting. The well-counter and PET/CT scanner were cross-calibrated as previously described by Vrist et al. (2). Whole blood and plasma data from our original study were reused for this study.

To convert measured activity from image-derived whole blood to plasma activity curves, plasma to whole blood activity ratios (PWR) were calculated for each of the samples.

In addition, all plasma values were transformed using the natural logarithm function. The slope and intercept of the resulting line at 40–60 and 40–90 mpi (plasma exponentials) were then determined by linear regression analysis and extrapolated back to the time for the peak. The plasma exponentials were used as a substitution for the corresponding image exponentials for the construction of the various input functions as described below. The 90-min samples were included for two reasons:

1. Interpolation of plasma data for calculation of *K_i_* at the time of whole-body (WB) data acquisition, instead of extrapolation.
2. Comparison of the *K_i_* results obtained using the 40–60 mpi plasma-curve substitution with results using the 40–90 mpi plasma-curve substitution, as in theory, the later sampling should result in a better fit to the original curve.

### 2.3. Image acquisition

The original PET/CT images were acquired on a Siemens Biograph mCT-4R 64 slice PET/CT scanner with a 22-cm axial field of view (FOV). The participants were positioned with the heart and the thoracic vertebrae Th7–Th10 centered in the FOV. After an intravenous bolus injection of 150 MBq ^18^F-NaF flushed with 20 ml isotonic saline, a 60-min list-mode dynamic scan was acquired immediately followed by a WB scan from the middle of the femur to the vertex of the skull acquired in 6–7 FOVs of 3 min per bed position.

### 2.4. Image reconstruction

The original PET images for dynamic analysis were re-binned into 50-time frames: 20 × 3 s, 12 × 5 s, 4 × 30 s, and 14 × 240 s. The reconstruction of PET scans used filtered back-projection with a Gaussian filter of 5 mm and a matrix size of 256 × 256 (3.2 mm × 3.2 mm × 1.4 mm). All dynamic images were automatically decay-corrected to the study injection time (study reference time). Image data from the WB scan were automatically decay-corrected to the start of the WB scan requiring additional decay correction to the study injection time for comparison with dynamic data.

Low-dose CT scans were performed, and the images reconstructed in three utilization-dependent series: (1) attenuation correction, (2) localization and identification of the thoracic vertebrae in the dynamic scan, and (3) localization of the relevant bone regions in the WB scan.

### 2.5. Image analysis

PMOD® version 4.206 software (PMOD Technologies LLC, Switzerland) was used for the non-linear regression analysis of the dynamic data and analysis of the static WB data.

### 2.6. Bone VOIs

**Figure 1:**
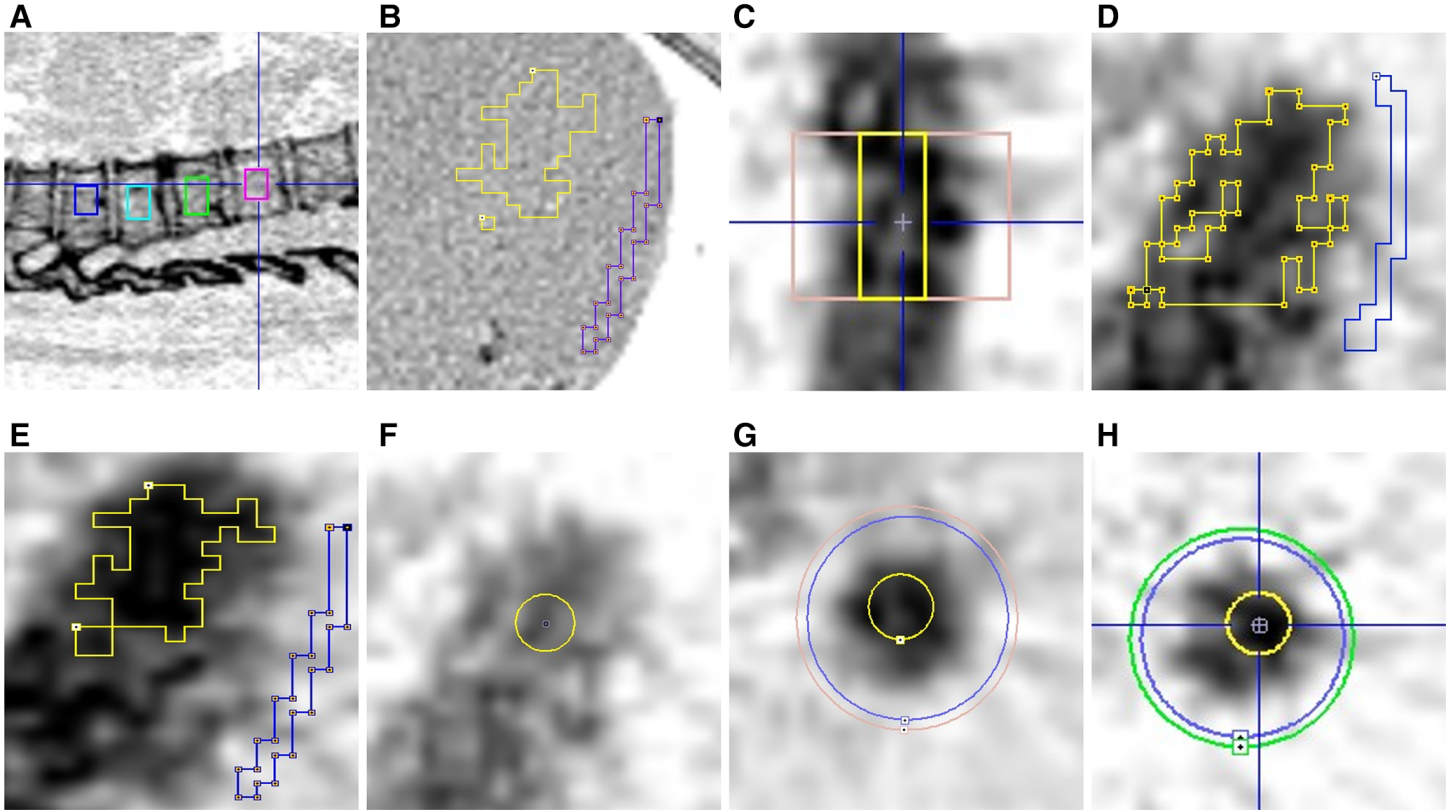
The various VOIs. (**A**) Vertebrae Th7-Th10; (**B**) CT-image–My-ocardial Bg (blue); (**C**) AO-Bg (pink); (**D**) LV-Orig (yellow); (**E**) LV-New (yellow); (**F**) LV-Fix (yellow); (**G**) AO-Fix (yellow); (**H**) AO-Peak (yellow).

For the reanalysis of the original data, all VOIs were constructed as described in the original study (2), whereas for the new optimization studies the vertebral VOIs (Th7/8–Th10/11) were drawn using a circular region of interest (ROI) in a single slice of 1-voxel thickness (1.4 mm) with a diameter of 4 voxels (12.8 mm), which was centered in the spongious bone and propagated through six slices resulting in a cylinder with a volume of approximately 1 ml in each of the four vertebrae (Figure 1A). Care was taken to avoid cortical bone and areas of obvious abnormal bone turnover (e.g., in a compressed vertebrae or for near lying large osteophytes). The data from the four individual VOIs were then unified to one combined VOI using the Union function in the PMOD VOI tools to improve counting statistics.

These combined VOIs were kept constant for each subject dataset and used throughout all studies of the various input functions described below.

### 2.7. Background VOIs

#### 2.7.1. LV studies

The background (myocardial Bg-VOI) was a ROI drawn on a single slice using the brush tool with a width of 1–3 pixels and propagation of the ROI over at least six slices. Its placement in the myocardial wall and as far as possible from the LV contour was controlled using the CT scan in the optimization studies (Figure 1B).

#### 2.7.2. Aorta studies

Two cylindrical VOIs were centered around the aorta at a level not lower than the middle of vertebrae Th7. The inner cylinder had a diameter (38–40 mm) approximately 2–3 voxels wider than the activity in the aorta and the outer cylinder had a diameter 4 mm wider than the inner cylinder. The lengths of the cylinders were 30–40 mm, depending on the curvature of the aorta and the necessity to avoid inclusion of, for example, the intercostal arteries, which might result in a false high background activity (Figure 1C). The background activity (*C*_Bg_) was calculated as the ratio between the differences in activity and the volume between inner and outer cylinders:

(1) $$C_{\mathrm{Bg}}=\frac{C_{\mathrm{In}}-C_{\mathrm{Out}}}{V_{\mathrm{Out}}-V_{\mathrm{In}}}(\mathrm{kBq}/\mathrm{ml}).$$

### 2.8. Input VOIs for image-derived input functions (IDIFs)

The VOIs for the IDIFs were constructed in five different ways. In the two studies using hot contouring of activity in the LV, the LV was delimited using a box VOI (approximately 40 mm × 40 mm × 40 mm). Within this VOI, a hot contour was drawn using PMOD's contour tool with the cutoff values described below.

The VOI definitions for these basic (unmodified) input functions were:

1. For reanalysis with original VOI definition (LV-Orig): a hot contour delineating 50%–70% of the max value in the box (Figure 1D). VOI volume: 50.8 ± 25.2.
  All contours were visually inspected for overlap with myocardial background and having a distance of at least two voxels (∼6.4 mm) from the myocardial background in all planes.
2. For LV-VOIs less prone to PVE and spill-over (LV-New): a hot contour delineating 60%–70% of the max value in the box (Figure 1E). VOI volume: 19.2 ± 11.3.
3. For LV-Fixed VOI studies (LV-Fix): A 12.8-mm diameter spherical VOI (∼4 voxels) was placed over the volume with highest activity in the center of the left ventricle (Figure 1F). VOI volume: 1.0 ± 0.6.
4. For the Aorta-Fixed VOI studies (AO-Fix): a cylindrical VOI with a diameter of 4 voxels (12.8 mm) was centered between the background VOIs described above, with the same length as the background VOI (Figure 1G). VOI volume: 3.4 ± 1.2.
5. For the Aorta-Peak VOI studies (AO-Peak): In the aorta a spherical peak-VOI (∼1 ml) was constructed with the peak VOI contouring tool using the inner background cylindrical VOIs as the delimiter in all frames (Figure 1H). VOI volume: 1.0 ± 0.6.

### 2.9. Correction of IDIFs for partial volume effect and spill-over from background

The basic input functions were derived from the activity data in the various input VOIs and converted to plasma activity by multiplying the whole blood activity with the PWR in all frames.

All basic input functions – LV-Orig, LV-New, AO-Fix, and AO-Peak with the exception of the LV-Fix – were corrected for PVE and Bg using the recovery coefficient RC_ß_ as described previously (2, 3):

(2) $$R_{\mathrm{IDIF}}(t)=RC_{\text{ß}}\cdot C_{A}(t)+(1-RC_{\text{ß}})\cdot C_{\mathrm{Bg}}$$

(3) $$\mathrm{such}\;\mathrm{that}\;RC_{\text{ß}}=(R_{\mathrm{IDIF}}(t)-C_{\mathrm{Bg}}(t))/(C_{A}(t)\text{–}C_{\mathrm{Bg}}(t)),$$

where *R*_IDIF_ is activity measured in VOIs in either LV or AO, *C*_Bg_ is background activity, and *C*_A_ is the “true” activity in arterial blood, which after 30 min equals the activity in venous blood (3).

For the comparison and estimation of the influence of background activity, all input data were also corrected using the simpler calibration factor RC_CF_ (1) without correction for spill-over from background activity:

(4) $$RC_{\mathrm{CF}}=R_{\mathrm{LV}}(t)/(C_{\mathrm{LV}}(t).$$

This equation can be used with VOIs with small volumes ≤1 ml, e.g., LV-Fix placed so far from surrounding background activity (>25 mm) that spill-over from background activity mostly can be ignored. An illustrative phantom measurement has shown that activity spill-over beyond this distance is rather constant and less than 8.2% (see Supplementary Section 1.5).

After correction, basic input functions were given identifiers by adding either -ß or -CF to the geometry nomenclature—e.g., “LV-VOI-Orig-ß” and so on.

### 2.10. Modification of the basic IDIFs

In all the basic image curves the terminal exponentials from 40 to 60 mpi were replaced by an exponential calculated from the plasma samples using either 40–60 mpi plasma exponentials or 40–90 mpi plasma exponentials.

All image values of the IDIFs were transformed using the natural logarithm function. The slope and intercept for the resulting line from 40 to 60 mpi (“terminal exponential”) was then determined by linear regression analysis and extrapolated back to the time for the peak.

The values of this line were subtracted from the values of the entire logarithmic curve to obtain the residual curve of the initial “fast image exponentials.”

The input curves were then reconstituted in two ways:

1. The logarithmically transformed plasma curve derived from the plasma samples (40–60 mpi and 40–90 mpi) were added to the logarithmically transformed residual curve and then exponentially retransformed as in our original study (2)—“the multiplicative logarithmic” (“Log”) method with the given identifiers “LV-Orig-Pl-40-90-Log (*t*_60_), and so on.
2. Both the logarithmically transformed residual curve and the plasma curve (40–60 mpi and 40–90 mpi) were retransformed using the exponential function before being added (6)—“the additive exponential” (“Exp”) method with the given identifiers “LV-Orig-Pl-40-90-Exp (*t*_60_),” and so on). The terminology “(*t*_60_)” indicates that the resulting *K_i_* values were obtained using the 60 mpi data point.

All input curve combinations used for calculating bone plasma clearance are presented in Supplementary Section 1.1 (Table S1).

### 2.11. Bone plasma clearance

*K_i_* ml/min^−1^·ml^−1^ was calculated as the mean value of four thoracic vertebrae (Th7–Th10 or Th8–Th11). No correction for delay was made as we found the delay to be of only a few seconds, and attempts at correcting the very noisy data in the first few acquisition frames failed to make the data more consistent.

### 2.12. Non-linear regression (NLR) analysis

The PMOD® version 4.206 software (PMOD Technologies LLC, Switzerland) was used to perform a two-tissue compartment dynamic NLR analysis of ^18^F-NaF-turnover as described by Hawkins et al. (8). The exchange of ^18^F-NaF between the compartments—plasma, extravascular, and bone—is described by the kinetic parameters *K*_1_-*k*_4_ and the parameter for regional bone plasma clearance *K_i_* is defined as

(5) $$K_{i}=\frac{K_{1\;}k_{3}}{k_{2}+k_{3}}$$

This NLR method was further analyzed for two values of the fractional blood volume vB, which was either fixed at 0.05 (vB-Fix) or determined by PMOD as a free-fitted (vB-Free) parameter.

### 2.13. Patlak analysis of dynamic studies

Assuming the efflux rate constant k_4_ to be negligibly small (*k*_4_ ≈ 0 min^−1^), the Patlak graphical analysis (9) provides a simpler alternative analysis method for measuring *K_i_* as described by equation 6 (5):

(6) $$\frac{C_{B}(T)}{C_{Pl}(T)}=K_{i}\frac{\int_{0}^{T}C_{Pl}(t)dt}{C_{Pl}\;(T)}+V_{0}$$

This equation approximates a straight-line fit with *K_i_* as the slope where *C_B_* and *C_Pl_* are the respective concentrations of tracer bound in bone and freely diffusible in plasma at each time point (*t*). V_0_ is the intercept of the ordinate and represents the apparent volume of distribution.

*K_i_* was calculated from the 60-min dynamic PET/CT scan using a bone TAC and each of the various IDIF modifications or selected semi-population input functions as described in Section 2.14. All Patlak results generated by the various input functions were compared to the basic corrected IDIF within each series and to the corresponding PMOD results.

### 2.14. Semi-population input functions (SPIFs) for static scan analysis

For the future analysis of static WB scans with the “best” input function as defined below, we derived an optimized three-exponential SPIF, as previously described by Blake et al. (6). The SPIF was derived from a population residual curve (PopRes) and then added to the terminal exponential derived from the plasma samples (described above under “Blood samples”), where the PopRes was derived from the corrected basic IDIFs scaled to a reference dose of 100 MBq, as used in the study by Puri et al. (1):

(7) $$\mathrm{SPIF}=\mathrm{PopRes}\;⊗\frac{\mathrm{Inj}\cdot \mathrm{dose}}{100}⊕({\mathrm{Plasma}}_{\mathrm{Exp}})$$

The residual curve represents the sum of the early fast exponentials and was derived by subtracting the terminal exponential (all data values ≥40 mpi) from the entire image-derived curve.

All residual curves were adjusted so that the times of peak count rate for all curves were coincident with the most frequent unadjusted peak time (16.5 s). The residual curves were then averaged to define the PopRes for combination with the plasma exponential to make the SPIF.

A mathematical model for the PopRes was fitted and is described in Supplementary Section 1.4.

### 2.15. Static scan analysis

As in the previous study (2), the static scan analysis was performed using a modified Patlak analysis with only two data points (4, 6). The first (0,*V*_0_) was obtained from either the original dynamic function or the reconstructed SPIF using the individual patient's blood samples. The second time point was obtained as the start time for the WB scan and the activity in the vertebrae (*C_B_*(*t*), *C_Pl_*(*t*)) at that time using the same VOIs as for the dynamic scan but adjusted for proper alignment. The values of the static scan data were corrected for decay to the time of injection/start of the dynamic scan for comparison with the dynamic data.

The *K_i_* values were then calculated as the slope of the line between (0,V_0_) and (*C_B_*(*t*), *C_Pl_*(*t*)) (4–6).

### 2.16. Statistics

The results are presented as mean ± standard deviation (SD) but, as the majority of the datasets obtained using the different input functions were not supposed to be normally distributed, visual representation of the data are presented in box-and-whisker plots showing Max, 75% quartile, Median, 25% quartile, and Min values as well as the difference (the box) between the 75% quartile and the 25% quartile (the interquartile range (IQR)).

Differences-between-method results were evaluated using Bland–Altman plots (10) showing the mean differences between the corresponding data points and the upper and lower 95% confidence limits.

Correlations between *K_i_* values obtained using different analysis models were calculated using Pearson's correlation coefficient but were not used as selection criteria when choosing the best agreement between the methods/parameter choices.

The percentage coefficient of variation of the PopRes curves was obtained as the ratio between the SD and the mean of the PopRes curve.

The PMOD χ^2^ test was used to evaluate the model fit of the input functions to the applied model.

The original and reanalyzed data were compared using a paired, two-tailed *t*-test.

## 3. Results

### 3.1. Reproducibility of original data

Comparison of the reanalyzed and original data (LV-Orig β series) using the original method definitions are summarized in Supplementary Section 1.2. The reanalyzed and original data (PWR, RC_ß_, AUCs of derived input curves, NLR-*K_i_* and Patlak-*K_i_* results) were all comparable.

The mean inter-observer difference for the NLR-ß-*K_i_* values were 0.0006 ± 0.0052, *r* = 0.91, and *p* = 0.72 (NS) and −0.0018 ± 0.0042. *r* = 0.89, and *p* = 0.17 (NS) for the Patlak-ß-*K_i_* values.

### 3.2. VOI volumes and corresponding recovery coefficients

The VOI volumes for input VOIs, Bg, and Bone with their corresponding RCs for the basic input IDIFs are shown in Table 1. The LV-Orig VOI has the lowest RC_ß_ (0.73 ± 0.17) compared with the AO-Fix VOI's RC_ß_ (0.99 ± 0.09). In contrast, the AO-Peak VOIs had RCs of 1.73 ± 0.62. The RC_CF_s were generally higher as spill-over was not included.

**Table 1:** VOI-volumes and correction factors for the basic IDIFs.

	PWR	Input-VOI ml	Bg VOI ml	Bone VOI ml	RC_ß_	RC_CF_
LV-Orig		48.7 (25.3)	8.00 (2.09)	8.37 (3.60)	0.73 (0.17)	0.88 (0.09)
LV-New		19.2 (11.3)	2.09 (0.65)	4.55 (1.66)	0.83 (0.13)	0.90 (0.08)
LV-Fix	1.16 (0.02)	1.0 (0.9)	4.51 (1.68)	4.51 (1.68)		0.95 (0.13)
AO-Fix		3.4 (1.2)	10.36 (3.94)	4.60 (1.73)	0.99 (0.1)	0.99 (0.09)
AO-Peak		1.0 (0.6)	10.22 (3.91)	4.60 (1.73)	1.77 (0.64)	1.77 (0.64)

[mean ± (SD)].

### 3.3. Comparison of the basic image derived input functions

All median and range data supporting the following observational results for the uncorrected and corrected Basic IDIF AUCs can be found in the Supplementary Section 1.3 (Figure S1A,B) box plots:

Correction of the basic IDIFs with either RC_ß_ or RC_CF_ tended to shift the AUCs toward higher AUC values (Supplementary Section 1.3, Figure S1B) but with less change in AUC values for smaller VOIs, the highest values being 377.53 ± 66.88 kBq·min and the lowest 293.57 ± 66.09 kBq min. The AO-Fix curves were almost unchanged while the AO-Peak curves showed lower values (*Δ*-AUC-mean: −187.1 ± 122.21 kBq min) with an unacceptably wide 95% CI of −426.6 to 52.5 kBq min.

### 3.4. Input functions with plasma-exponential substitutions

The effect of substituting the final image exponential in the corrected basic IDIFs with plasma exponentials is shown in the box plots (Supplementary Figure S2A–D).

IDIFs that were modified using the exponential additive method generally had a lower IQR and range for the 40–90 mpi data analysis compared with the corresponding 40–60 mpi analysis curves (*Δ*-IQR: −33.6–1.8; range: −7.7 to −19.7). However, the mean AUCs for the LV input functions were not significantly different from the corresponding basic input functions as shown in Supplementary Section 1.3 (Table S3). This also applied to the SPIFs reconstructed from the LV-New-ß series. The small differences in AUCs between the SPIFs and their corresponding IDIFs were not significant.

**Figure 2:**
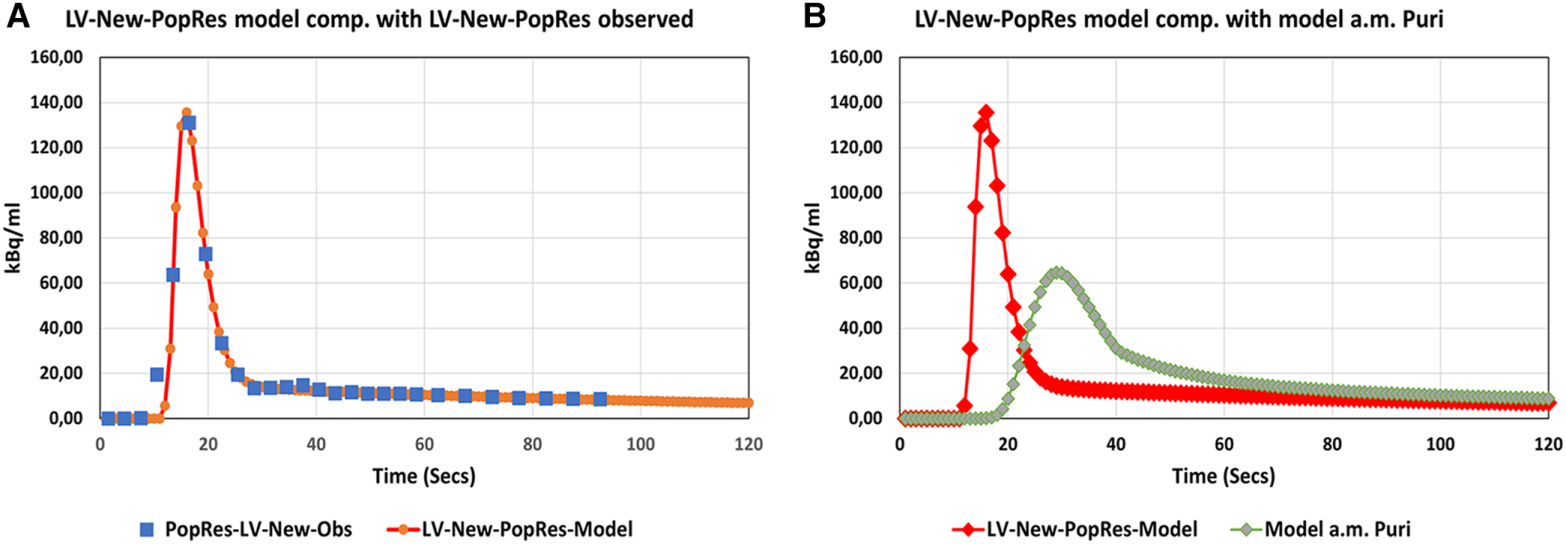
Population residual curves. (**A**) Modeled Pop-Res curve (red) compared with observed data (blue squares). (**B**) Modeled Pop-Res curve (red) compared with Pop-Res model (green) constructed using parameters published by Puri et al. (1).

In Figure 2A, the model of our PopRes was compared with data from the observed, optimized PopRes curve. The visual fit was very good and use of the curve results in comparable *K_i_* results, as described in the following subsections. The mean difference was −0.40 ± 2.79 with a 95% CI of −5.87 to 5.06. The AUC_1800sec_ was 4,078 kBq s.

The mathematical best-fit curve for our PopRes is presented in Supplementary Section 1.4.

### 3.5. *K_i_* results using the various input functions

#### 3.5.1. Patlak analysis

**Figure 3:**
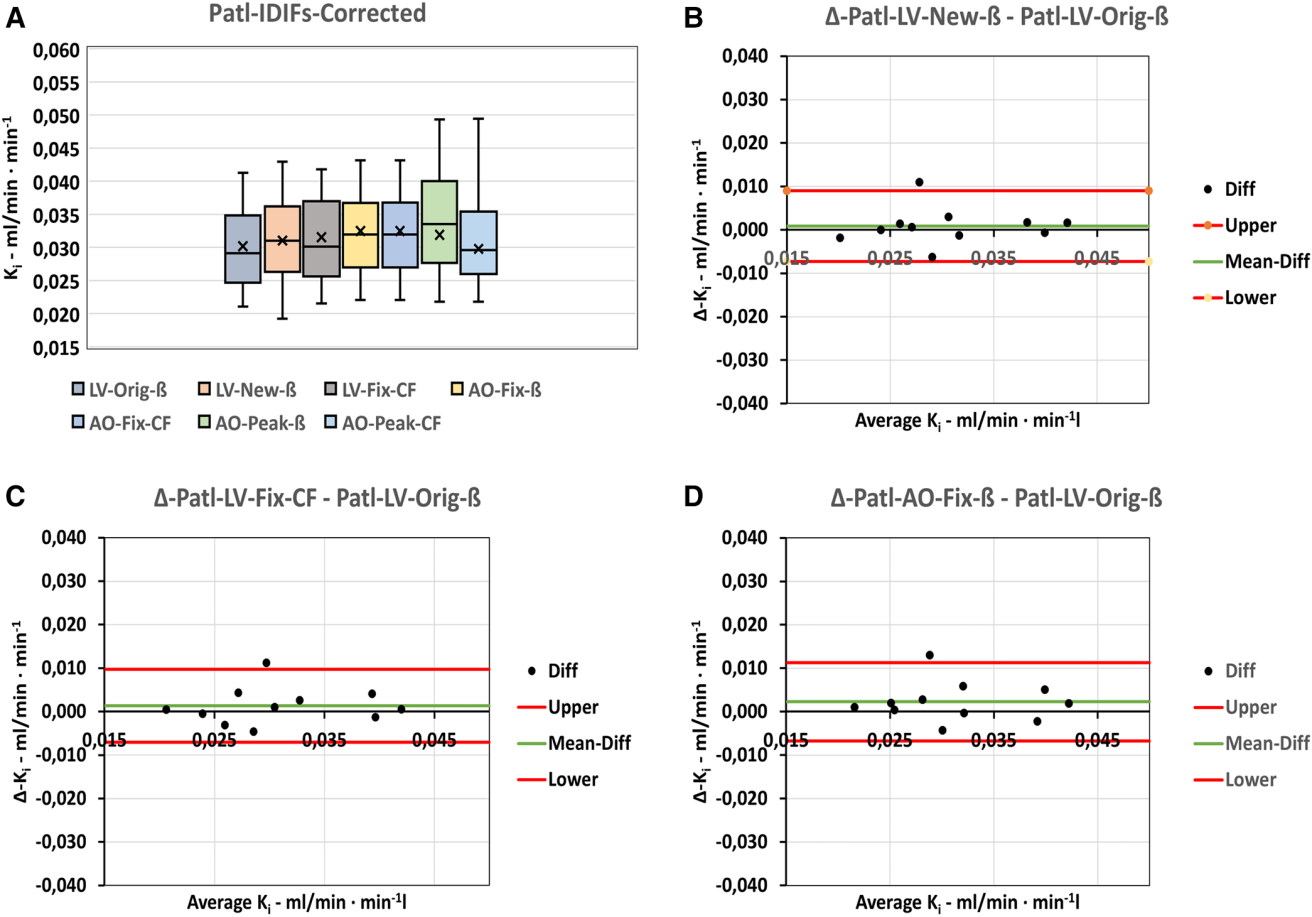
Cross comparison of Patlak-*Ki*-results using the basic corrected IDIFs. (**A**) Box-plot shoving the distribution of data within the various series. (**B–D**) Bland-Altman plots of the differences between the basic LV-Orig-ß input function the basic input functions most comparable to LV-Orig-ß. “Upper” and “Lower” refers to 95%-confidence limits.

All Patlak results for the various corrected, basic IDIFs were compared as illustrated in Figure 3A–D. The box plot in Figure 3A shows the distribution of data for all the input series and their values in Table 2.

**Table 2:** Dynamic Patlak-*Ki*-results using the basic and modified IDIFs.

IDIF-Corrected	LV-Orig-ß	LV-New-ß	LV-Fix-CF	AO-Fix-ß	AO-Peak-ß
Basic	0.0302 (0.0071)	0.0310 (0.0073)	0.0315 (0.0072)	0.0325 (0.0070)	0.0319 (0.0120)
PL-40-60-Log	0.0304 (0.0075)	0.0321 (0.0071)		0.0315 (0.0081)	0.0319 (0.0120)
PL-40-90-Log (*t*_60_)	0.0300 (0.0073)	0.0312 (0.0069)		0.0321 (0.0077)	0.0306 (0.0076)
PL-40-90-Log (*t*_90_)	0.0316 (0.0071)	0.0327 (0.0068)		0.0333 (0.0076)	0.0320 (0.0105)
PL-40-60-Exp	0.0300 (0.0072)	0.0317 (0.0070	0.0306 (0.0095)	0.0319 (0.0077)	0.0334 (0.0100)
PL-40-90-Exp (*t*_60_)	0.0316 (0.0071)	0.0311 (0.0070)	0.0314 (0.0070)	0.0322 (0.0075)	0.0324 (0.0079)
PL-40-90-Exp (*t*_90_)	0.0317 (0.0070)	0.0326 (0.0070)	0.0326 (0.0070)	0.0334 (0.0074)	0.0336 (0.0094)

[mean ± (SD), ml/min min^−1^].

Results for the LV-New-ß and LV-Fix-CF (Figure 3A) show comparable median and mean values, whereas the LV-Orig-ß are a little lower and the AO-Fix-ß and AO-Fix-CF a little higher, but the observed differences are not significantly different from zero, as shown in the corresponding Bland–Altman plots (Figure 3B–D). The data in Table 3A show the LV-New-ß to have the smallest confidence interval and the least mean difference and variance compared with the LV-Orig-ß function. However, apart from the AO-Peak results, which show an unacceptable large variance (Figure 3A), the differences and variation were generally small.

**Table 3:** Bland-Altman-analysis of Patlak-*K_i_*-results.

	Mean-Diff	SD	CL_UL_	CL_LL_	CI
A. Compared with LV-Orig-ß					
LV-New-ß	0.0009	0.0042	0.0090	−0.0073	0.0164
LV-Fix-CF	0.0013	0.0043	0.0097	−0.0070	0.0168
AO-Fix-ß	0.0023	0.0046	0.0113	−0.0067	0.0180
B. Compared with basic IDIFs					
LV-Orig-Pl-40-40-90-Log	−0.0020	0.0011	0.0017	−0.0020	0.0037
LV-New-Pl-40-40-90-Log	0.0002	0.0011	0.0023	−0.0019	0.0043
AO-Fix-Pl-40-40-90-Log	−0.0004	0.0014	0.0023	−0.0031	0.0054
LV-Orig-Pl-40-40-90-Exp	−0.0001	0.0006	0.0011	−0.0014	0.0025
LV-New-Pl-40-40-90-Exp	0.0002	0.0008	0.0018	−0.0013	0.0031
LV-Fix-Pl-40-40-90-Exp	−0.0001	0.0018	0.0034	−0.0037	0.0071
AO-Fix-Pl-40-40-90-Exp	−0.0006	0.0014	0.0012	−0.0025	0.0037

CL_UL_ and CL_LL_, upper and lower 95% confidence limit; CI, confidence interval.

All results in ml/min ml^−1^.

(A) Differences between the selected basic Patlak-IDIFs and LV-New-ß. (B) ifferences between the selected basic Patlak-IDIFs and LV-New-ß.

**Figure 4:**
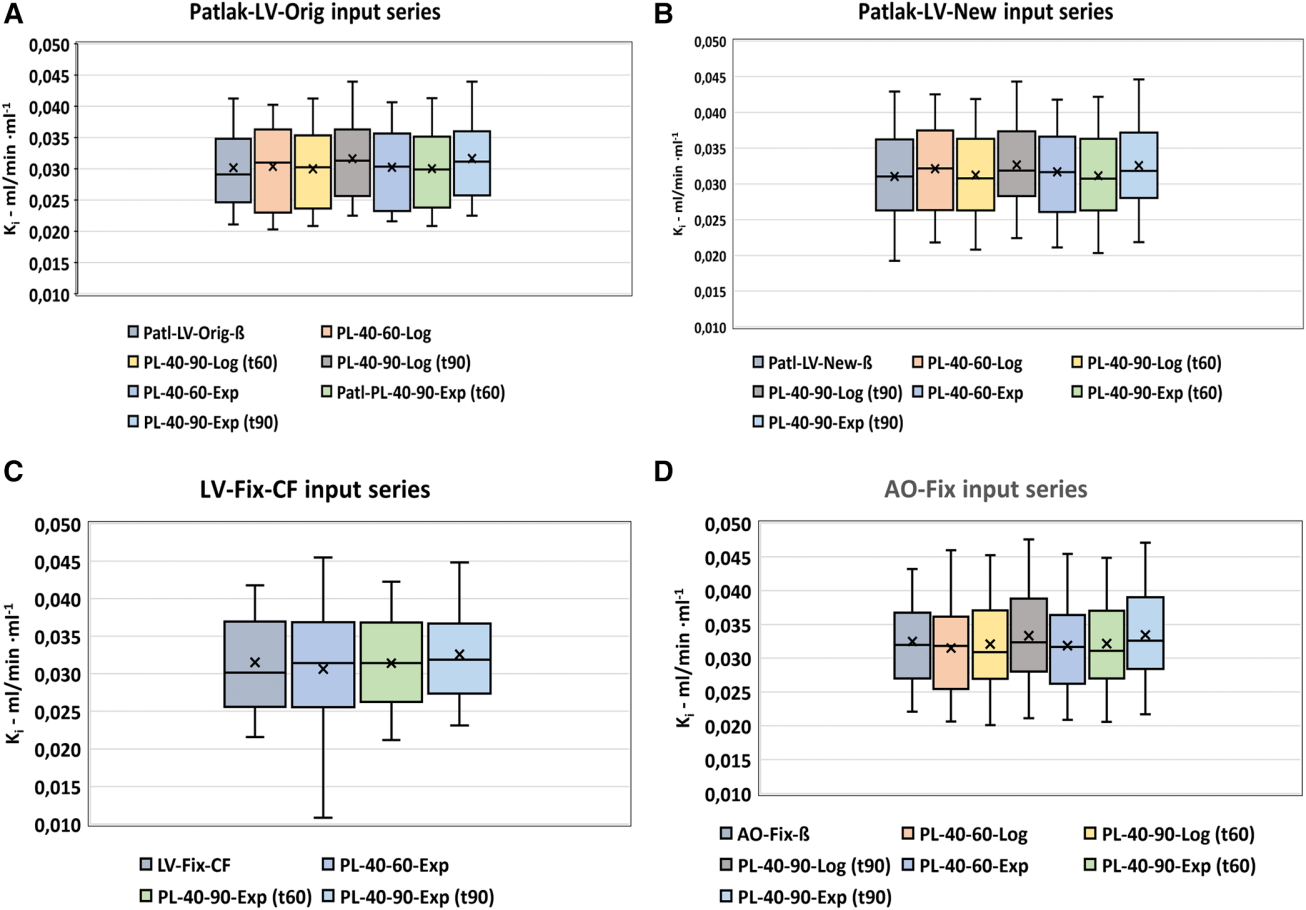
Patlak analysis. Serial comparison of the various modifications of the basic IDIFs. Box-plots (**A–D**) shows the distribution of values using the various input functions.

In order to select the derived input function with the least difference and variance compared with the corresponding corrected basic input function, the Patlak data were compared serially as shown in Figure 4 and in Supplementary Section 2.1 (Figure S3). In all series, the *K_i_* results at *t* = 60 mpi substitution with plasma exponentials 40–90 mpi using the exponential method showed the least difference and variation compared with the basic corrected IDIF, with the smallest values observed for the LV-New-ß series compared with the LV-Original method (Table 3B).

For all input functions, the mean differences between the use of the exponential (additive) method and the logarithmic (multiplicative) method are found to be very small, but the smallest variance is observed for the use of the exponential method (Table 3B).

#### 3.5.2. Non-linear regression (NLR) analysis

For completeness, all NLR-fitted kinetic parameter results for the differing input analysis methods are found in Supplementary Section 2.2 (Table S4A,B).

The mean *K_i_* values obtained for the various corrected, basic IDIFs were determined using either a constricted vB-Fix of 0.05 (Supplementary Table S4A) or calculated as part of the PMOD parametric fitting. The *K_i_* values using free-fitted vB resulted in higher *K_i_* values for all five input functions (Supplementary Table S4B).

**Table 4:** NLR-analysis using the various IDIFs.

*Ki*	LV-Orig-ß	LV-New-ß	LV-Fix-CF	AO-Fix-ß	AO-Peak-ß
Fix vB	0.0406 (0.0111)	0.0378 (0.0112)	0.0397 (0.0111)	0.0432 (0.0095)	0.0603 (0.0374)
Free vB	0.0424 (0.0115)	0.0408 (0.0111)	0.0422 (0.0117)	0.0450 (0.0102)	0.0625 (0.0377)
Mean-Diff	0.0180 (0.0013)	0.0030 (0.0031)	0.0025 (0.0033)	0.0017 (0.0024)	0.0022 (0.0035)

*Ki*-Mean ± (SD), ml/min ml^−1^. Upper row: Fixed-vB = 0.05. Middle row: Free-fitted vB. Lower row: Mean-Diff between the two modes.

**Figure 5:**
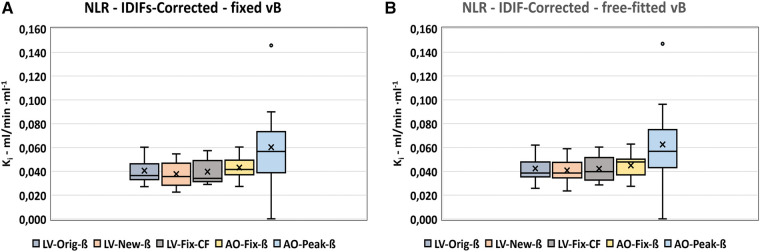
NLR-*Ki*-results using fixed vB vs. free-fitted vB. (**A**) NLR-corrected, vB = 0.05. (**B**) NLR-corrected, free-fitted vB.

The distribution of *K_i_* values using the corrected five basic input functions are shown in Figure 5A,B. The results are comparable except for the AO-Peak-IDIF, which shows the largest difference and variance and hence was excluded from further analysis.

The differences between *K_i_* values using the various input functions and LV-Orig-ß (Supplementary Section 2.2, Figures S4A–F and Supplementary Table S5) were not significant but the confidence intervals were rather variable with the smallest variance found for the LV-New-ß with vB-free-fit (Supplementary Figure S4D). The LV-Fix input function showed the largest confidence interval in both modes.

The vB-Free had a mean value of 0.01 ± 0.008 across the input modes.

#### 3.5.3. Comparison of Patlak results with NLR results

**Figure 6:**
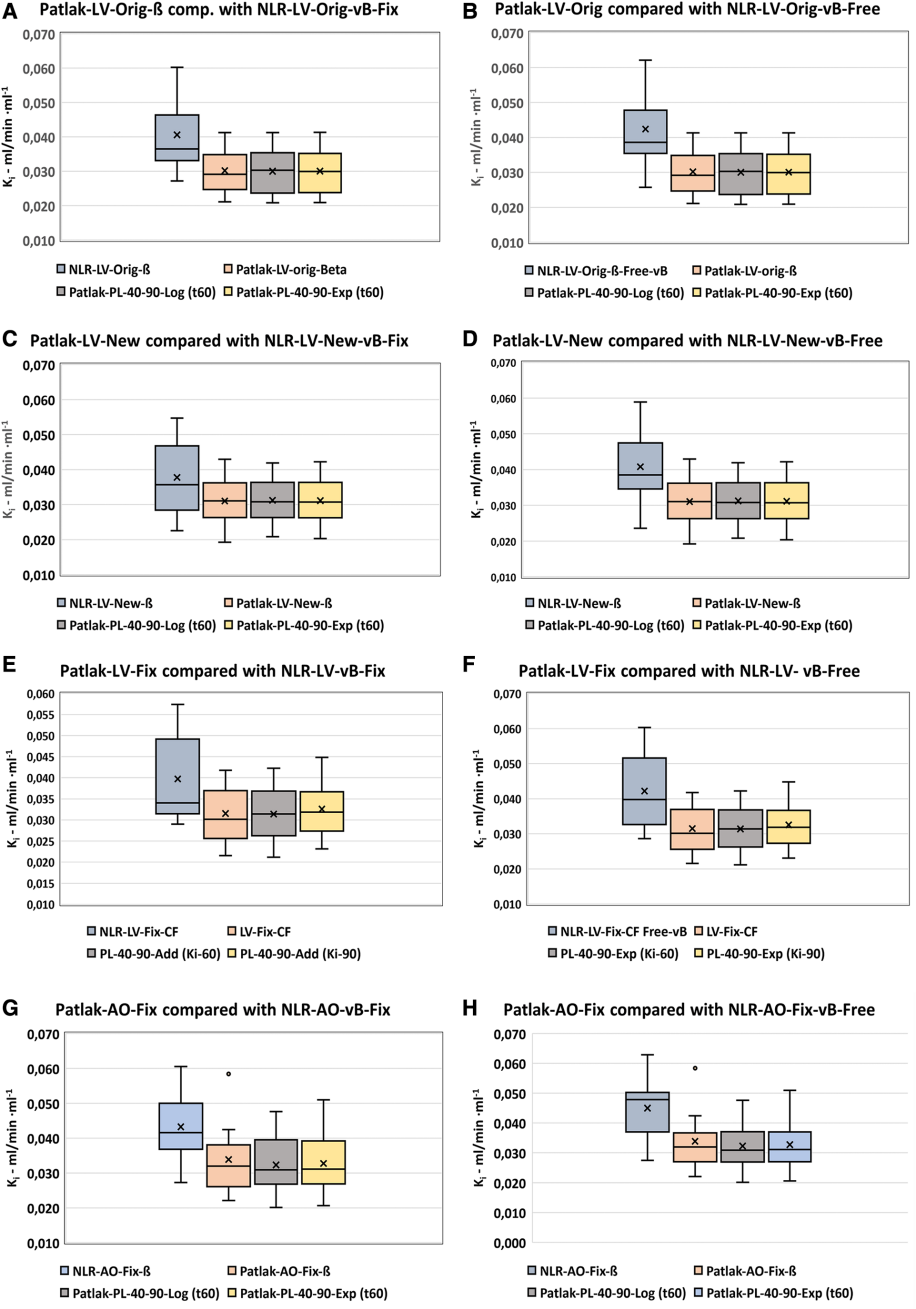
Distribution of optimized NLR-Ki-values compared with the corresponding Patlak-values. (**A**,**B**) LV-Orig-ß. (**C**,**D**) LV-New-ß. (**E**,**F**) LV-Fix-CF. (**G**,**H**) AO-Fix-ß. All analyses with either fixed vB (left panels) or free-fitted vB (right panels).

Based on the prior analyses (Sections 3.5.1, 3.5.2), the NLR-*K_i_* results for the LV-Orig-ß series, LV-New-ß series, and AO-Fix-ß series were chosen for comparison with the corresponding Patlak-*K_i_* results, with the distribution of data shown in Figure 6A–H and the corresponding Bland–Altman plots in Supplementary Section 2.3 (Figure S5A–H). Quantitative results are presented in Table 5 and the mean differences in Supplementary Section 2.3 (Table S6). The smallest difference and confidence interval are seen with the LV-New-ß series and, as such, this series is identified as our choice of optimum analysis method. All observed variances are comparable with *Δ*-*K_i_* ≤ 0.013 ml/min· min^−1^ (Supplementary Section 2.3, Table S6).

**Table 5:** Comparison of NLR- (fixed and free vB) and Patlak- *K_i_*-analysis for selected basic input functions and their derivatives.

	NLR-IDIF-Fix-vB	NLR-IDIF-Free-vB	Patl-IDIF	Pat–PL-40-90-Log	Pat-PL-40-90-Exp
LV-Orig-ß	0.0406 (0.0111)	0.0424 (0.0115)	0.0302 (0.0071)	0.0300 (0.0073)	0.0300 (0.0072)
LV-New-ß	0.0378 (0.0112)	0.0408 (0.0111)	0.0310 (0.0073)	0.0312 (0.0069)	0.0311 (0.0070)
LV-Fix-CF	0.0397 (0.0111)	0.0422 (0.0117)	0.0315 (0.0072)	0.0314 (0.0070)	0.0326 (0.0070)
AO-Fix-ß	0.0432 (0.0095)	0.0450 (0.0102)	0.0339 (0.0101)	0.0323 (0.0082)	0.0327 (0.0087)

*Ki*-Mean (SD), ml/min ml^−1^.

#### 3.5.4. Comparison of dynamic results with results using semi-population functions

**Figure 7:**
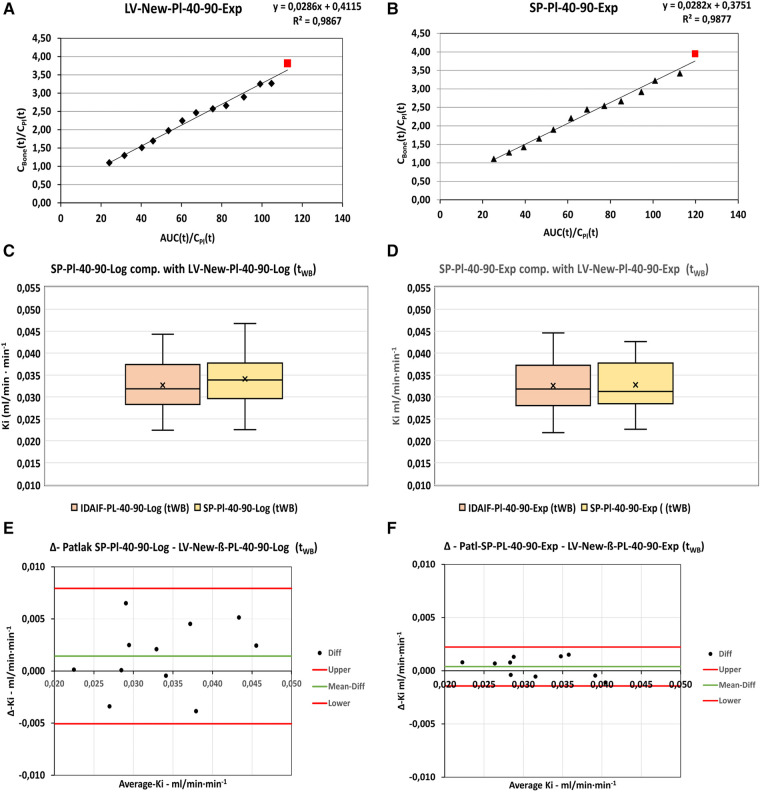
Examples of static scan data (red square) plotted in combination with dynamic data (black triangles) for the same patient using (**A**): Patl-LV-New Pl-40-90-Exp input function and (**B**): SP-New-Pl-40-90-Exp input function. (**C**,**D**): Comparison of Ki-results obtained by the dynamic input functions and SP-input functions using the logarithmically substituted and the exponentially substituted IDIFs, respectively. (**E**,**F**): Bland-Altman analysis of the corresponding logarithmically and exponentially substituted IDIFs. “Upper” and “Lower” refers to 95%-Confidence limits.

For this analysis, we exclusively used data for the LV-New-ß-Input series (optimum analysis) and the semi-population functions derived from these (Figure 2A). In Figure 7A,B, Patlak-analysis plots from the same patient are shown using the LV-New-Pl-40-90-Exp and the corresponding SP-Pl-40-90-Exp input functions. In both plots, data from the static scan are shown as a red square lying close to the regression line. The distribution of data using dynamic input functions are shown in the box plots in Figure 7C,D with quantitative differences shown in the Bland–Altman plots of Figure 7E,F, for the Log (multiplicative) and Exp (additive) analysis methods, respectively. The observed differences within the dynamic series were smallest for the Exp series, as seen in Figure 7F.

#### 3.5.5. Static scan analysis using two-point Patlak analysis

**Figure 8:**
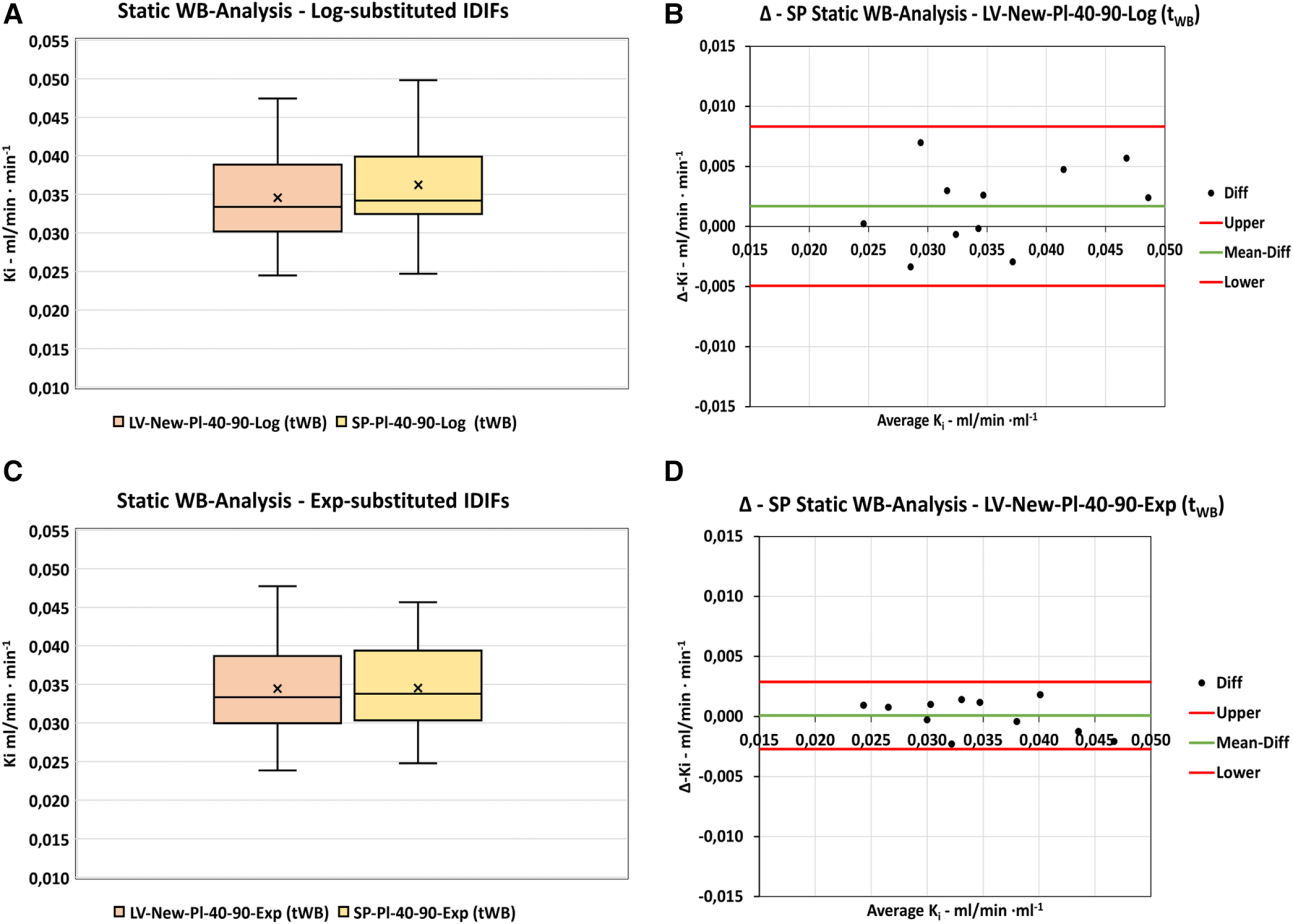
Static WB-scan analysis. Comparison of *Ki*-results obtained by dynamic LV-New-Pl-40-60 functions and the corresponding derived SP-input functions using Patlak Two-Point analysis. (**A,B**) Distribution of data and differences using logarithmically substituted IDIFs. (**C,D**) Distribution of data and differences using exponentially substituted IDIFs. “Upper” and “Lower” refers to 95%-confidence limits.

The results for the static scan analyses using the two-point Patlak analysis with either the LV-New-Pl-40-90 dynamic input functions or the derived SPIF are shown in Figure 8A–D and Table 6. As for the dynamic input functions, the series modified using exponential substitution of the final exponentials showed the lowest mean *K_i_*-difference from zero with the 95% confidence interval of −0.0027 to 0.0029.

**Table 6:** Patlak analysis.

	LV-New-Pl-40-90-Log	SP-Pl-40-90-Log	LV-New-Pl-40-90-Exp	SP-Pl-40-90-Exp
Dyn-Patl	0.0327 (0.0068)	0.0341 (0.0077)	0.0326 (0.0070)	0.0328 (0.0063)
Stat-Patl	0.0346 (0.0071)	0.0362 (0.0083)	0.0345 (0.0073)	0.0345 (0.0067)

Upper row: Comparison of dynamic results using either IDIFs or semi-population functions at t_WB_: 66 ± 2 mpi. Lower row: Results from static scan analysis using static scan “two-point”-Patlak-analysis.

*Ki*-Mean (SD), ml/min ml^−1^.

Compared to the dynamic results presented above, the *K_i_* values measured using the static scan analysis were 0.0019 ± 0.0017 ml/min ml^−1^ higher than those for the corresponding dynamic scan analysis. However, all differences were well within the 95% confidence limits (−0.0015 to 0.0053 for both the logarithmic and exponential series) and thus were not significantly different from zero.

## 4. Discussion

### 4.1. Comparison with original results

As a test of inter-observer reproducibility, all original image data (2) were reanalyzed using the original method parameters (Section 3.1). The observed slight difference (NS) in the plasma-to-whole-blood ratio (PWR-reanalyzed = 1.16 ± 0.02. vs. PWR-Orig = 1.17 ± 0.03) lies within the range of values presented in Figure 3 in Vrist et al. (2) and is most likely due to the more selective exclusion of plasma outliers in the reanalysis in the present study.

The RC_ß_ correction factor used in the present study (0.72 ± 0.17) was comparable to that of the original study (0.69 ± 0.15).

The AUC of the basic input curves in this study is 374 ± 59 kBq min, which is comparable with the 353 ± 59 kBq min result from the original study, with the resulting *K_i_* values of the NLR and Patlak analyses differing slightly, but with an acceptable inter-observer difference (NLR results: 1.3%; Patlak results: 6.3%).

Reproducible values between the original data analysis of this and the original study (2) are a prerequisite for being able to attribute subsequently observed differences in kinetic parameters as being due to this study's use of “optimized” input functions while keeping all other parameters (PWR, Bg-VOIs, and Bone-VOIs) almost constant in all series.

This assumption is supported by previous repeatability studies on the use of ^18^F-NaF-PET to evaluate SUV as a measure for uptake in cancer patients (11, 12), as well as ^18^F-NaF-PET being previously used successfully for studies on changes in *K_i_* values before and after treatment of osteoporosis (4, 7, 13).

### 4.2. Effects on basic input functions by VOI size and correction factors

Comparing the VOI volumes and their corresponding RCs, it is obvious there is a correlation between these parameters, as the RCs are derived from the activity in the VOIs and compared with the actual blood sample activities (Table 1): the smaller the VOI, the closer to 1 the correction factor, except for the AO-Peak VOI in which the factors are much higher than 1. This correlation has been described previously in cardiac FDG-PET studies (14) and can be ascribed to a combination of PVE and an increasing mean activity (kBq/ml) in the VOIs as the smaller volumes are placed, more selectively, over areas with the highest activity.

The RC_ß_ corrects the activities measured in the input VOI for both PVE and Bg (3), whereas the RC_CF_ only corrects for PVE. The RC_ß_ (0.99 ± 0.09) and RC_CF_ (0.99 ± 0.09) are identical for the AO-Fix-ß VOIs, indicating the background in and around the aortic wall is negligible, at least at this level of the thoracic aorta. These factors are comparable to the coefficients reported in a study by Puri et al. (15). However, for the AO-Peak VOIs, the correction factors are unrealistically high (1.73 ± 0.63) and result in a much larger variance in the derived *K_i_* results when compared with the other corrected input functions.

As the correction factors were primarily lower than 1, the general effect on the uncorrected input functions was to increase the AUCs as shown in Supplementary Figure S1. The closer the correction factor is to 1, as in the AO-Fix curves, the lesser the shift in values.

The exception to this was the AO-Peak curves which, due to the correction factors of 1.73 ± 0.63, resulted in smaller AUCs and larger variance. The most likely reason for the poor AO-Peak VOI performance is due to its inherent poor counting statistics caused by the small VOI volume of only a few voxels in combination with the amount of activity injected and the short time resolution in the initial dynamic acquisition bins.

### 4.3. Effect of substituting the final exponentials of basic input functions with plasma exponentials

The general effect of substituting the final image exponentials with plasma exponentials was smaller IQRs, as seen in the box plots in Supplementary Figure S2. However, the AUC mean differences were not significantly different across the series of input functions (Supplementary Section 1.3, Table S3).

The substitution methods producing AUC results (kBq/ml min) closest to the basic corrected IDIFs were the LV-New-Pl-40-90-Log (*Δ*-Mean: −1.17, 95% Cl −17.7 to 15.3) and the LV-New-40-90-Exp (*Δ*-Mean: 3.2, 95% Cl −9.7 to 16.2). For comparison, the corresponding values for the AO-Fix-40-90-Exp were much higher (*Δ*-Mean: −11.0, 95% Cl −44.4 to 22.5; *p* = 0.04).

### 4.4. Comparison of *K_i_* results using the various analysis methods

#### 4.4.1. Patlak analysis

The Patlak analysis was applied to all basic IDIFs in order to find the IDIFs with the least variances of *K_i_* values, after which they were compared with the results using the basic LV-Orig-ß-IDIF. Our choice for comparing to LV-Orig-ß-IDIF was not because we believe our original analysis method to be “a reference standard” but simply to assess the performance of this method compared with the others (“comparative reference”).

From the box plot in Figure 3A, the LV-New-ß, LV-Fix-ß, and AO-Fix-ß are identified as all being qualified methods. From the Bland–Altman plots in Figure 3B–D and the data in Table 3, it is shown that no IDIF showed *K_i_* results significantly different from those of the basic LV-Orig-ß-IDIF but the LV-New-ß IDIF resulted in the least difference when compared with the LV-Orig-ß-IDIF (0.0009 ± 0.0042 ml/min ml^−1^).

The modified IDIF with the least variance and least difference of *K_i_* values compared with the basic corrected IDIF was selected from a serial comparison of the *K_i_* values obtained by the modified IDIFs as shown in Figure 4, Tables 2 and 3, and in Supplementary Section 2.1 (Figure S3).

Of all series, the LV-New-IDIFs with plasma substitution 40-90-Exp showed the least variance, with the AO series showing the highest variances, but the mean differences between the modified IDIFs and the corresponding basic IDIFs were not significantly different from zero.

The *K_i_* results of the input functions obtained using small VOIs tend to be a little higher than the LV-Orig series but this may simply be due to these functions having smaller AUCs, which, in turn, results in a higher ratio between activity uptake in the bones compared with the lower activity in the plasma. Generally, as can be seen in Figure 3A and Tables 2 and 3, the variations in *K_i_* across the various basic and derived input functions are small, but even so, the *K_i_* mean difference of 0.0023 ± 0.0046 ml/min ml^−1^ for the Patl-AO-Fix-ß-IDIF compared with Patl-LV-Orig-ß was significantly higher than that of Patl-LV-New-ß (*p* = 0.01).

Overall, our data are comparable with results reported in other studies. In the original study by Hawkins et al. (8) of thoracic vertebrae in normal individuals using image-derived input functions, the mean *K_i_* value was 0.093 ± 0.0071. In a study by Frost et al. (16) of hemodialysis patients suspected for adynamic bone disease and osteoporosis, the mean *K_i_* results in the lumbar spine were 0.028 ± 0.012 and 0.027 ± 0.005, respectively. Thus, for example, the mean basic Patlak results for our patients (0.0302 ± 0.0071 to 0.0325 ± 0.0070) are well within this rather wide range.

#### 4.4.2. Non-linear regression (NLR) analysis

The NLR analysis based on the Hawkins model has often been used as a reference standard for the less complicated Patlak analysis (2, 8, 17, 18). In our original study, we used the PMOD Software® with the default vB of 0.05, which is a value previously reported by Messa et al. (19). However, it has been questioned why we did not allow the vB to be fitted freely; therefore, we have examined the possible differences using both NLR-Fix vB = 0.05 and NLR-Free vB as shown in Figure 5 and Table 4 and in Supplementary Section 2.2 (Figure S4 and Table S5).

The general effect on *K_i_* values using the free-fitted vB tended to be 3.7%–8.0% higher values (5.3% on average). However, none of these differences were significantly different from zero, but the AO-Peak-ß showed a very high SD of 0.35 ml/min ml^−1^ with a CV of approximately 60% compared with 27% for the other IDIFs. Based on this observation, we excluded this variant from further studies, as it would probably be too insensitive for the detection of small changes in *K_i_*.

The NLR-Free-fit vB analysis showed a smaller range of *K_i_* values, especially for the LV-input series (Supplementary Section 2.2, Table S4), and this mode will be chosen for future dynamic study analyses. This is in accordance with the results reported by Puri et al. (20), showing the CV for *K_i_* to be approximately 15% whereas the *K_1_–k_4_* parameters had CVs of at least 30%, which is likely caused by the NLR method being sensitive to statistical noise in the input data.

In this study, we found the averaged free-fitted value for vB to be 0.01 ± 0.008. This is about one-fifth of the value of 0.05 originally reported by Messa et al. (19) and five times the value of 0.002 used in more recent studies by Puri et al. (21). We have no explanation for these differences, but differences between bone regions and disease populations seem plausible.

#### 4.4.3. Comparison of Patlak results with NLR results

The variances of the NLR results were much higher than the variances of the Patlak results. As discussed in the previous subsection, this is probably due to the NLR method having a greater sensitivity to statistical noise in input data, whereas the Patlak analysis appears to be very robust, as indicated by the relatively small differences observed across the various input series (Table 3).

It is well known that Patlak results are generally lower than NLR results, which have previously been reported to be −7% by Brenner et al. (17), −13% by Installé et al. (22), and −23.7% by Puri et al. (23).

In the present study, the differences were in the range of −17.3% to −26.1% for the NLR-Fix vB and −22.8% to −29.2% for the NLR-Free fit vB, with the smallest difference being for the LV-New-ß series in both comparisons (Supplementary Section 2.3, Table S6). In our original study, we found a difference of −17.4 ± 10% (2), so the optimized data are within the previously reported range.

Consequently, our aim was to select the Patlak series showing the least variance and difference when compared with the PMOD-NLR analysis, both with fixed vB and free-fitted vB as shown in Figure 6 and Supplementary Section 2.3 (Table S6, Figure S5).

#### 4.4.4. Comparison of dynamic results with results using semi-population functions

Based on the above results and discussion, we chose to derive our optimized population residual functions from the LV-New-Pl-40-90-Exp input function, which was least variable and least different from the corresponding basic input data. This population residual was used to construct the semi-population curves to be used as input functions for the static scan analysis as originally described by Frost et al. (4) and further used in, for example, studies by Vrist et al. (2) and Blake et al. (6).

As shown in Figure 2A, the parameters for our model CKD-MBD population residual curve result in a good fit with the curve derived as an average of the residuals obtained from our analyses. Compared with an osteoporosis population model curve calculated using the parameters recently published by Puri et al. (1) (Figure 2B), there are some differences in peak height and full-width-half-maximum (FWHM) of the peaks, even though our data were normalized to the injected dose of 100 MBq as used in the study by Puri et al. (1). As a result, the AUC_1800s_ of our curve (4,078 kBq s) was approximately 12% lower than that of 4,560 kBq s reported by Puri et al. (1). The reason for the observed differences in the curve forms is probably due to “delay and dispersion,” which is to be expected as our input function were obtained from the left ventricle, whereas Puri et al. (1) used the abdominal aorta as input for the study of bone metabolism in lumbar vertebrae. In comparison, our aorta input curves at the level of Th7 showed no significant difference in shape or peak height, with a delay of only 3 s compared with those of the left ventricle.

However, apart from the technical issues regarding the curve differences, the interesting question of whether these differences are caused by differences in bone metabolism in different patient populations (1, 7) cannot be conclusively answered using the data from this study or our original study, as we were unable to use identical VOI definitions and placement in the arterial system. The data show that differences in the placement of input VOIs matters but, despite this, the AUCs of the two different population residual curves only differ by approximately 12% without corrections. Thus, the use of an “universal input model” for various anatomical regions and/or patient groups may be feasible but should be done with great care and possibly with the inclusion of a relevant correction for delay and dispersion (24) in order for the derived results to be comparable between patient groups. For studies of serial changes in bone plasma clearance within the same patient, this is probably less important.

The resulting *K_i_* values for the semi-population functions showed no significant difference for either the Log series or the Exp series, but the variance and 95% confidence interval for the differences were much smaller for the Exp series. Thus, this input function derivation will be chosen for future studies as this should make the detection of smaller differences possible when comparing serial measurements of *K_i_*, such as previously described by Frost et al. (4) before and after treatment with teriparatide, compared to use of a semi-population function with a wider variance and confidence interval.

#### 4.4.5. Static scan analysis

In this study the LV-New input functions showed no significant difference between *K_i_* values obtained using the dynamic input function or the corresponding SPIF-Exp series, but a little higher value was obtained using the SPIF-Log series (Table 6). The results are comparable to the original dynamic results but with a little lower *K_i_* value compared to our original static scan *K_i_* result of 0.0395 ± 0.011 ml/min ml^−1^. This may be explained by the AUC of our original SPIF (331 ± 70 kBq min^−1^) being on average 9% smaller than our optimized AUC of 362 ± 47 kBq min, thus resulting in a higher *K_i_* value.

## 5. Conclusions

We have established that our original logarithmic/multiplicative method is valid, producing quantitative results comparable to the exponential/additive method, but with the results having a little more variance.

We have shown that recovery coefficients/correction factors are correlated to the volume of input VOIs and their location and that the correct choice of correction depends on the applied analysis geometry.

Within limits, the corrected input functions have comparable AUCs and yield correspondingly comparable *K_i_* results but with differing variances.

The use of a blood sample taken at 90 mpi, corresponding to the end of the entire ^18^F-NaF-PET study protocol after the static data acquisition and not just at the end of the dynamic data collection, improves the fit between the original IDIF and the IDIF with the final exponential substituted by the plasma exponential using blood samples between 40 and 90 mpi.

Even though differences between the *K_i_* results obtained using the various input functions are small and comparable, we recommend the use of the same analysis implementation technique for future comparative studies due to the possibility of the differing variances making serial changes in *K_i_* more difficult to detect for the use of non-optimized analysis parameters/methods.

A comparison of two models for population residual curves for differing patient populations showed significant differences in peak shape (peak height and FWHM), but a difference of approximately 12% only in total AUC, which indicates that a comparison of data may be possible, but the input curves should ideally be obtained with comparable anatomical input VOIs or at least corrected for differences in delay and dispersion. To answer the question of whether the input curves vary due to changes in bone metabolism and/or between various patient population studies would require a comparative study utilizing the same analysis technique in order to avoid methodological pitfalls.

## Data Availability

The original contributions presented in the study are included in the article/Supplementary Material, further inquiries can be directed to the corresponding author.
