# Supplementary material for: A systematic evaluation of five different image-derived input functions for the clinical implementation of 18F-NaF bone PET/CT in patients with chronic kidney disease–mineral and bone disorder
Source: Front Nucl Med. 2023 Jul 28;3:1235800. doi: 10.3389/fnume.2023.1235800 (PMC11440843; doi:10.3389/fnume.2023.1235800)
Supplement: Supplementary file 1 [file Datasheet1.pdf]

Supplementary material

1 Supplementary input data.

1.1 Definitions of input functions

The simplest input-functions were basic IDIFs without or with correction for PVE and spill-over. IDIFs used for calibration with blood samples were derived by substituting the final image values with plasma exponentials using either the logarithmic-multiplicative- (Log) or exponential-additive methods (Exp) calculated from either 40 – 60 mpi or 40 – 90 mpi plasma samples. LV – left ventricle, AO – aorta.

Table 1. Schema of the basic input functions and their derived input functions.

Basic IDIFs	Correction-Mode	Method for plasma-exponential substitution	Time period for plasma-exponential (mpi)
LV-Orig, LV-New	$RC_{ß}$	Logarithmic (Log)	40-60
			40-90
		Exponential (Exp)	40-60
			40-90
LV-Fix	RC_{CF}	Exponential (Exp)	40-60
			40-90
AO-Fix, AO-Peak	$RC_{ß}$	Logarithmic (Log)	40-90
			40-90
		Exponential (Exp)	40-60
			40-90
	RC_{CF}	Logarithmic (Log)	40-60
			40-90
		Exponential (Exp)	40-60
			40-90

1.2 Reproducibility of original data

The results of the reanalyzed data using the original method parameters (2) were compared with the original results as presented in Table 2.

Table 2. Inter-observer variation: Comparison of reanalyzed and original data

	Reanalyzed	Vrist et al 2021 (2)
**Plasma/whole blood ratio**	1.16 ± 0.015	1.17 ± 0.03
**RC*ß***	0.72 ± 0.17	0.69 ± 0.15.
**AUC- LV-Orig-ß-Pl-40-60(t_{60}) kBq·min**	359 ± 76	353 ± 59
**AUC-LV-Orig-Pl-40-90-Log (t_{60}) kBq·min**	376 ± 64	341 ± 54
**NLR-ß-Ki ml/min · ml^{-1}**	0.0420 ± 0.0117	0.0415 ± 0.0125
**Patlak-ß-Ki ml/min · ml^{-1}**	0.0319 ± 0.009	0.0337 ± 0.091

1.3 AUC of the optimized basic and derived input functions

Figure 1. A: AUC (kBq · min) of basic uncorrected IDIFs. B: AUC of corrected basic IDIFs.

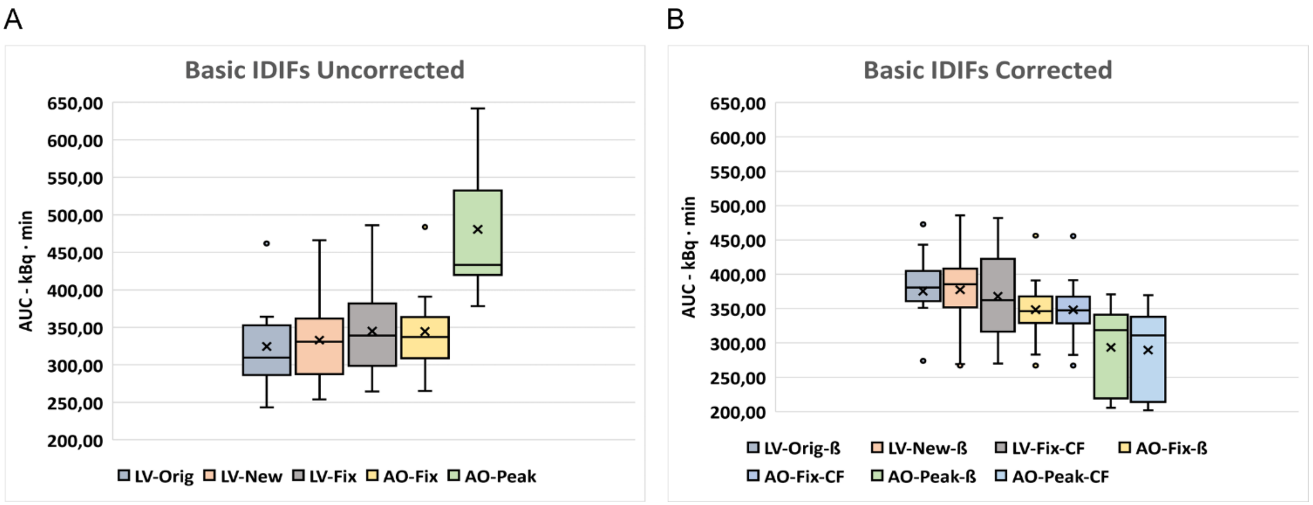

Figure 2. AUC of corrected IDIFs with substitution of terminal exponential 40-60 and 40-90 mpi. Effect of using plasma samples of either 40-60 mpi or 40-90 mpi. A: Using the logarithmic method or B: Using the exponential method.

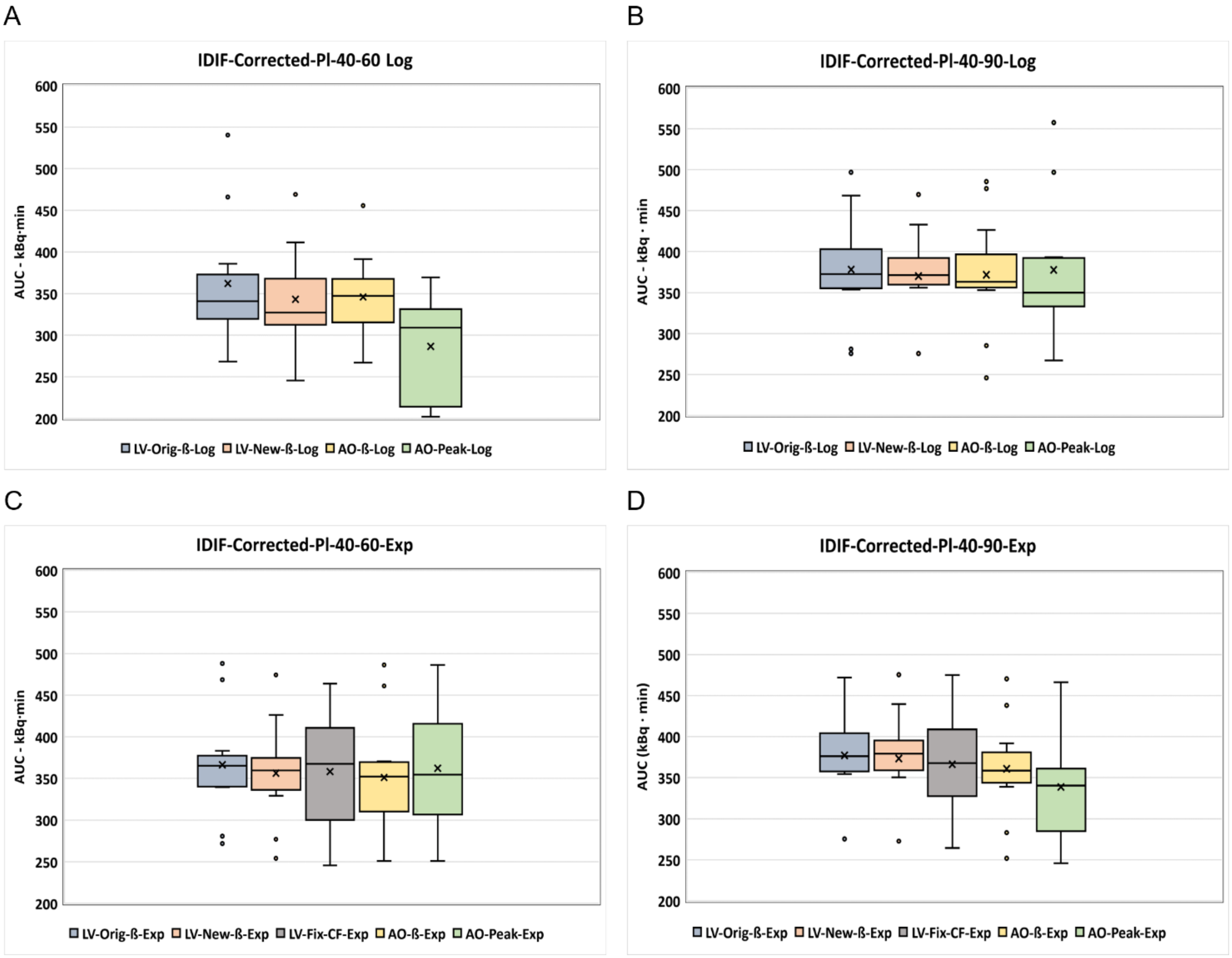

Table 3. AUC Mean ± (SD) kBq · min of the corrected basic and derived input functions.

	LV-Orig-ß	LV-New-ß	LV-Fix-CF	AO-Fix-ß	AO-Peak-ß	SPIF-LV-New
**Basic Input Corr**	375.58 (61.13)	377.53 (66.88)	367.72 (74.73)	348.40 (51.36)	293.57 (66.09)	
**Pl-Korr-40-90-Log**	378.24 (67.19)	370.13 (57.32)		371.66 (71.48)	377.66 (82.98)	367.29 (81.68)
**Pl-Korr-40-90-Exp**	377.07 (63.67)	373.33 (60.50)	366.20 (64.58)	360.71 (61.28)	338.61 (67.17)	362.31 (49.07)

1.4 Population residual curve model

The population residual curve was derived from the LV-New-Pl-40-90-Exp curves and a mathematical model fitted to the observed data using the PMOD-application. The best-fit population-residual curve (Article Figure 2A) was a gamma-function with 2-exponentials able to model its high and narrow input peak:

$$C_{Cont}(t) = \begin{cases} 0, & t < Begin \\ [A_1 u^B - A_2 - A_3] e^{-u\, ln(2)/T_1} + A_2 e^{-u\, ln(2)/T_2} + A_3 e^{-u\, ln(2)/T_3}, & t \geq Begin, u = t - Begin \end{cases} \quad \text{(Eq. 8)}$$

The best-fit parameters for the modelled (CKD-BMD) PopRes curve scaled to a reference dose of 100 MBq were:

Parameter	Value	Unit
Begin	12.3	s
B	2.0	
A_1	79.610563	kBq/ml
T_1	0.09554	min
A_2	12.071616	kBq/ml
T_2	1.179091	min
A_3	3.108064	kBq/ml
T3	8.853892	min
Chi^2-value	22.0	

1.5 Activity quantification: Phantom data

Recovery coefficient from NEMA IQ phantom data (ideal geometry)

Sphere-activity: 18 MBq; Bg-activity: 1.8 MBq; Sph-Act : Bg-Act.: 10:1

Sphere Ø (mm)	VOI Ø (mm)	VOI vol (cm3)	RC_B
37.0	31.8	16.9	0.89
37.0	25.4	8.5	0.97
28.0	19.1	4.0	0.94

Spillover (myocardial representative geometry)

Spill-over was measured in a simple phantom using a 60 ml syringe with activity vertically suspended in a water filled beaker (with no activity). Activity was measured in concentric cylinders 2 pixels wide (6.4 mm). The activity measured in rings 4-6 (25.6 – 38.4 mm from center) were constant (35.6 kBq/cm3) corresponding to a value of 8.2% of the radioactive concentration in the syringe (sum of activity in Ring 1 and 2: 432.6 kBq/cm3). The vertical blue lines represent the inner and outer wall of the syringe. Each data point represents the activity within the border between 2 adjacent cylinders.

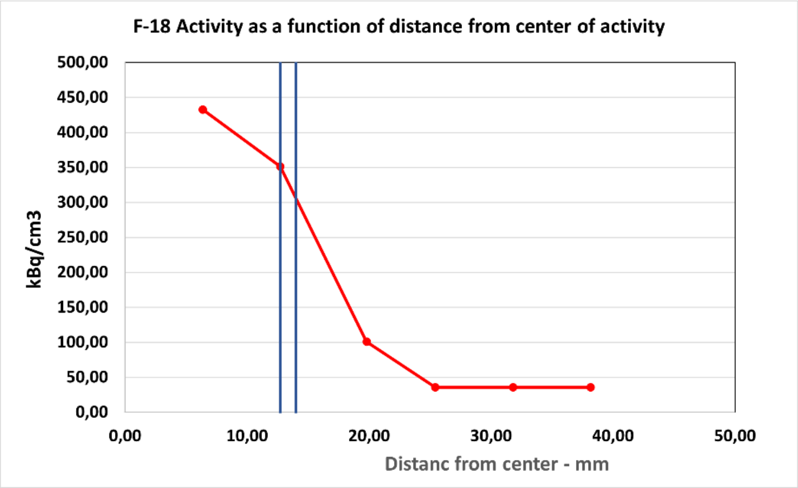

2. Supplementary Ki-results

2.1 Dynamic Patlak analysis

Figure 3. A-D Serial Patlak analysis. Bland-Altman plots showing differences between the basic IDIFs and the Pl-40-90-Log-IDIFs and the Pl-40-90-Exp-IDIFs.

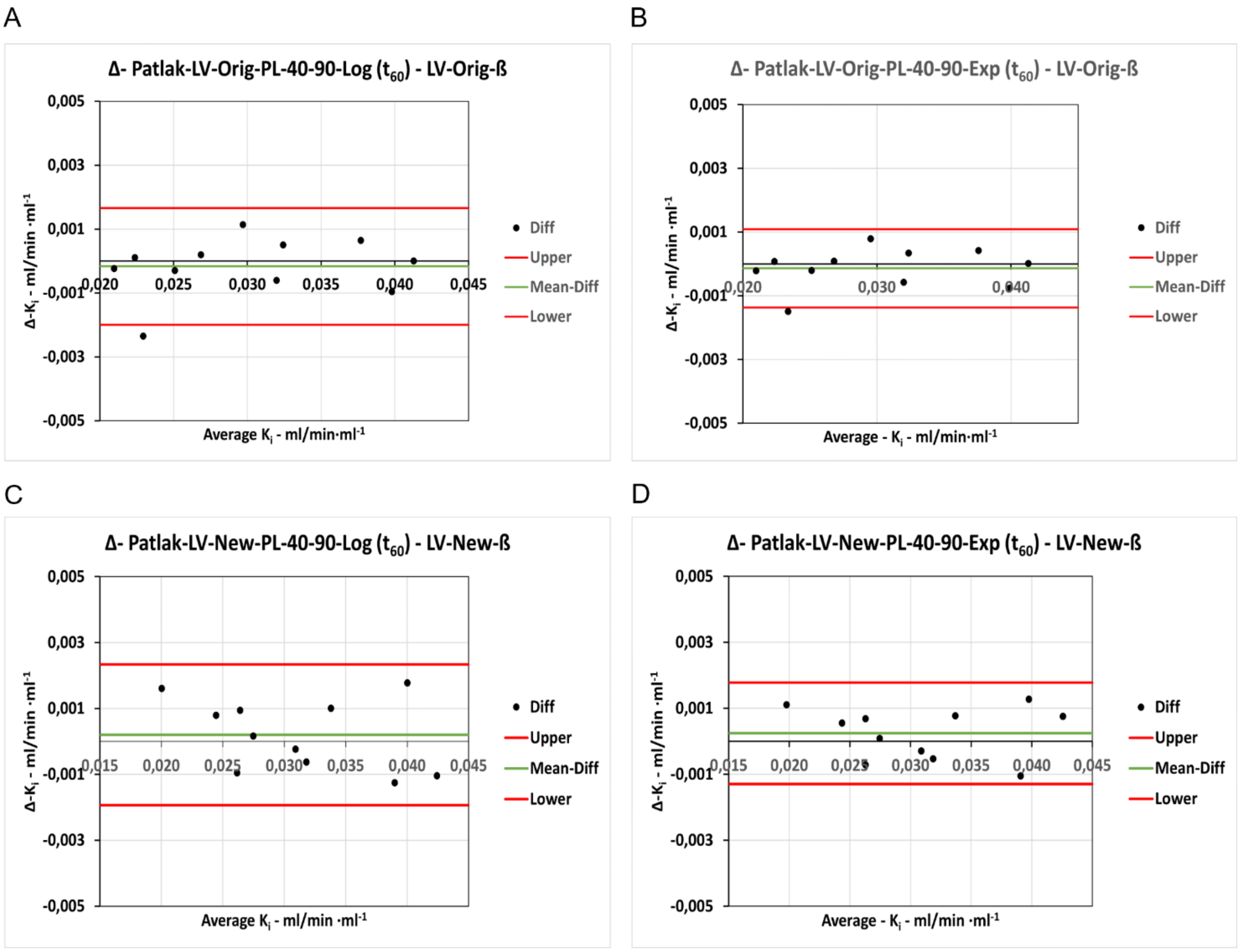

Figure 3. E-H Serial Patlak analysis. Bland-Altman plots showing differences between the basic IDIFs and the Pl-40-90-Log-IDIFs and the Pl-40-90-Exp-IDIFs. There are no Log-data for the LV-Fix-series (E).

E

No Log-data

F

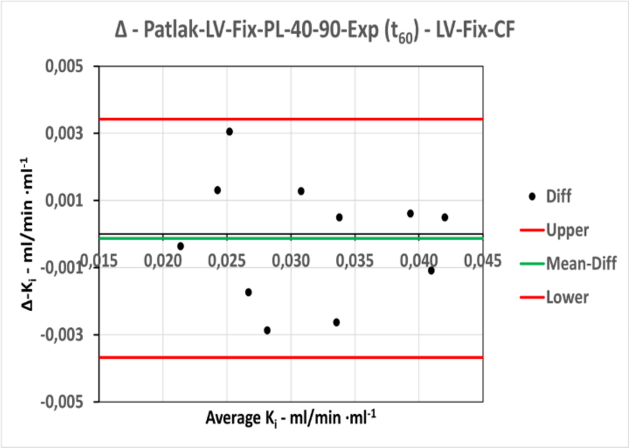

G

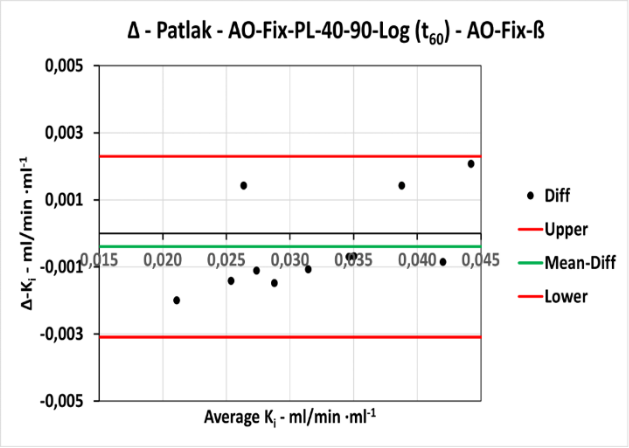

H

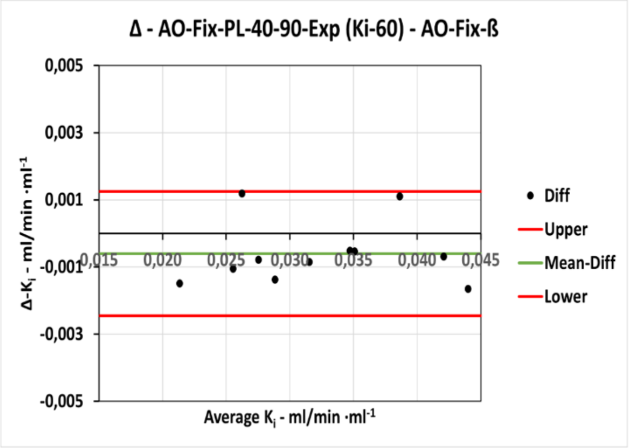

2.2 NLR-results using the various input functions

Table 4. All NLR- results. Mean ± (SD) (A): vB = 0.05. (B): Free-fit vB

A	Fix-vB	K1	k2	k3	k4	K_1/k_2	Flux	Chi^2
**LV-Orig-ß**	0.05	0.1045 (0.0422)	0.2719 (0.1421)	0.1708 (0.0369)	0.0105 (0.0073)	0.4477 (0.1930)	0.0406 (0.0111)	41.9 (24.8)
**LV-New-ß**	0.05	0.0930 (0.0400)	0.2215 (0.1824)	0.1469 (0.1022)	0.0066 (0.0053)	0.6364 (0.3886)	0.0378 (0.0112)	56.7 (39.3)
**LV-Fix-CF**	0.05	0.0977 (0.0404)	0.2451 (0.1733)	0.1721 (0.1305)	0.0085 (0.0060)	0.5274 (0.3787)	0.0397 (0.0111)	56.9 (35.4)
**AO-Fix-ß**	0.05	0.1221 (0.0682)	0.3116 (0.2577)	0.1801 (0.1026)	0.0096 (0.0046)	0.5064 (0.2257)	0.0432 (0.0095)	45.1 (30.0)
**AO-Peak-ß**	0.05	0.1310 (0.0642)	0.3266 (0.3206)	0.2874 (0.2224)	0.0361 (0.0454)	1.3357 (2.0617)	0.0603 (0.0374)	47.5 (29.8)

B	Free-vB	K1	k2	k3	k4	k1/k2	Flux	Chi2
**LV-Orig-ß**	0.0048 (0.0065)	0.1702 (0.0579)	0.8446 (0.1907)	0.2852 (0.0476)	0.0109 (0.0056)	0.2066 (0.0645)	0.0424 (0.0115)	25.1 (21.3)
**LV-New-ß**	0.0096 (0.0139)	0.1490 (0.0606)	0.7002 (0.3240)	0.2677 (0.0990)	0.0085 (0.0054)	0.2332 (0.0749)	0.0408 (0.0111)	34.2 (25.0)
**LV-Fix-CF**	0.0088 (0.0133)	0.1599 (0.0578)	0.7801 (0.4387)	0.2734 (0.1197)	0.0099 (0.0057)	0.2420 (0.0906)	0.0422 (0.0117)	34.8 (25.1)
**AO-Fix-ß**	0.0157 (0.0167)	0.1849 (0.0967)	0.8091 (0.5582)	0.2651 (0.1176)	0.0099 (0.0046)	0.2728 (0.0972)	0.0450 (0.0102)	32.3 (23.9)
**AO-Peak-ß**	0.0106 (0.0129)	0.2135 (0.1323)	0.9399 (0.6457)	0.4171 (0.2288)	0.0205 (0.0162)	0.2430 (0.0886)	0.0625 (0.0377)	32.7 (24.0)

Figure 4. Bland-Altman analysis showing the mean-differences ± 95%-confidence limits between NLR-Ki-results using the various ß- or CF-corrected IDIFs compared with LV-Orig-ß. Results were obtained with either fixed vB (0.05) (left column) or free-fitted vB (right column).

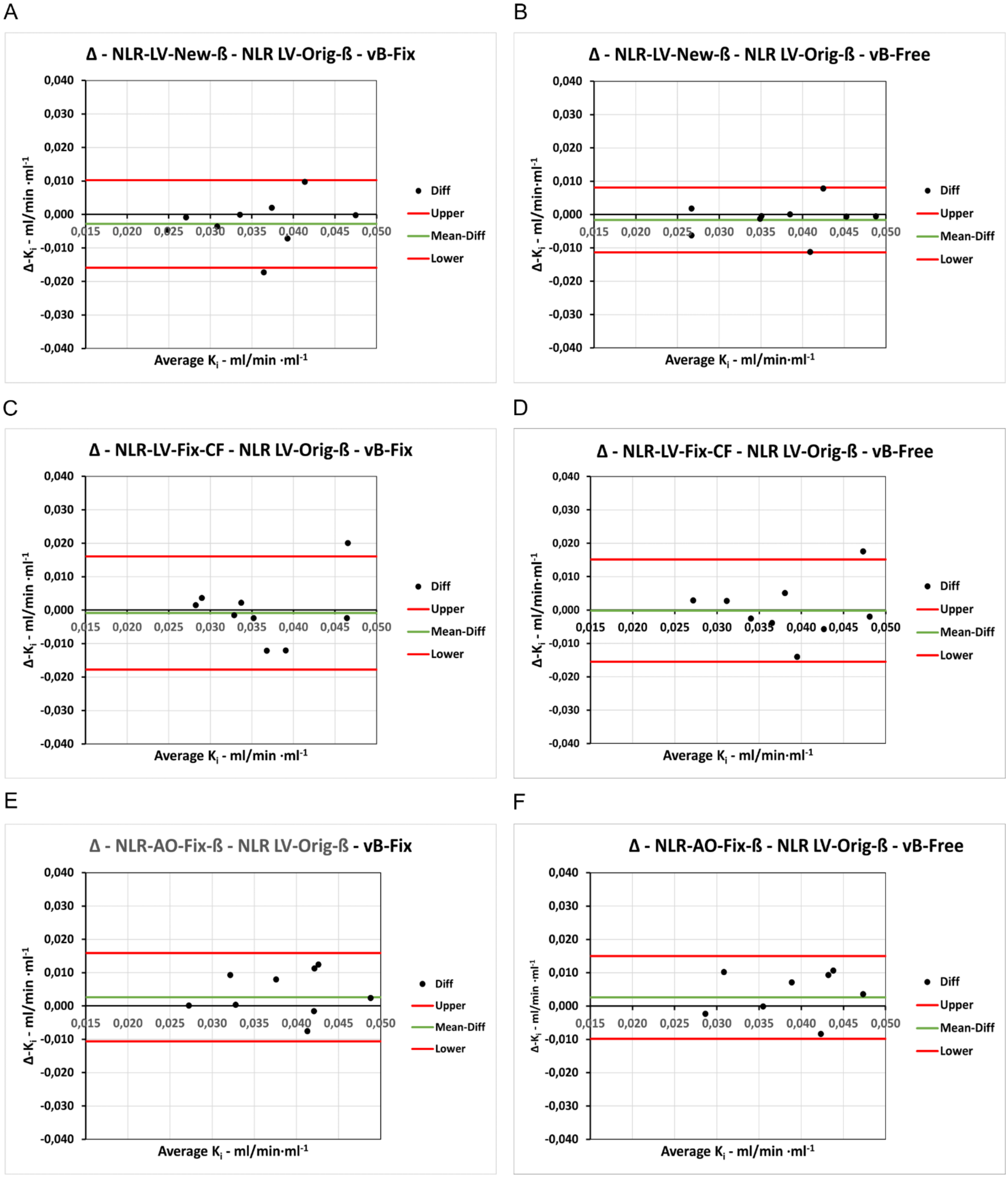

Table 5. Bland-Altman analysis of NLR-K_i-results. Differences between the selected IDIFs with their corresponding NLR-LV-Orig-ß analysis with either fixed vB or free-fitted vB. All Results in K_i ± SD ml/min·ml^{-1}. CL_{UL} and CL_{LL}: Upper and lower 95%-confidence limit. CI: Confidence interval.

NLR-Fix vB	Mean-Diff	SD	CL_{UL}	CL_{LL}	CI
**LV-New-ß**	-0.0028	0.0067	0.0103	-0.0159	0.0262
**LV-Fix-CF**	-0.0009	0.0086	0.0160	-0.0178	0.0338
**AO-Fix-ß**	0.0026	0.0068	0.0159	-0.0106	0.0265
**AO-Peak-ß**	0.0197	0.0360	0.0902	-0.0508	0.1410

NLR-Free vB	Mean-Diff	SD	CL_{UL}	CL_{LL}	CI
**LV-New-ß**	-0.0016	0.0050	0.0081	-0.0113	0.0194
**LV-Fix-CF**	-0.0002	0.0078	0.0151	-0.0155	0.0307
**AO-Fix-ß**	0.0026	0.0063	0.0150	-0.0098	0.0248
**AO-Peak-ß**	0.0201	0.0326	0.0840	-0.0437	0.1276

2.3 Comparison of NLR and dynamic Patlak K_i-results

Figure 5 (A-D). Comparison of optimized NLR-Ki-values with the corresponding Patlak-values. A and B: the LV-Orig-ß. C and D: LV-New-ß. E and F: AO-Fix-ß IDIFs with either fixed vB (left panel) or free-fitted vB (right panel).

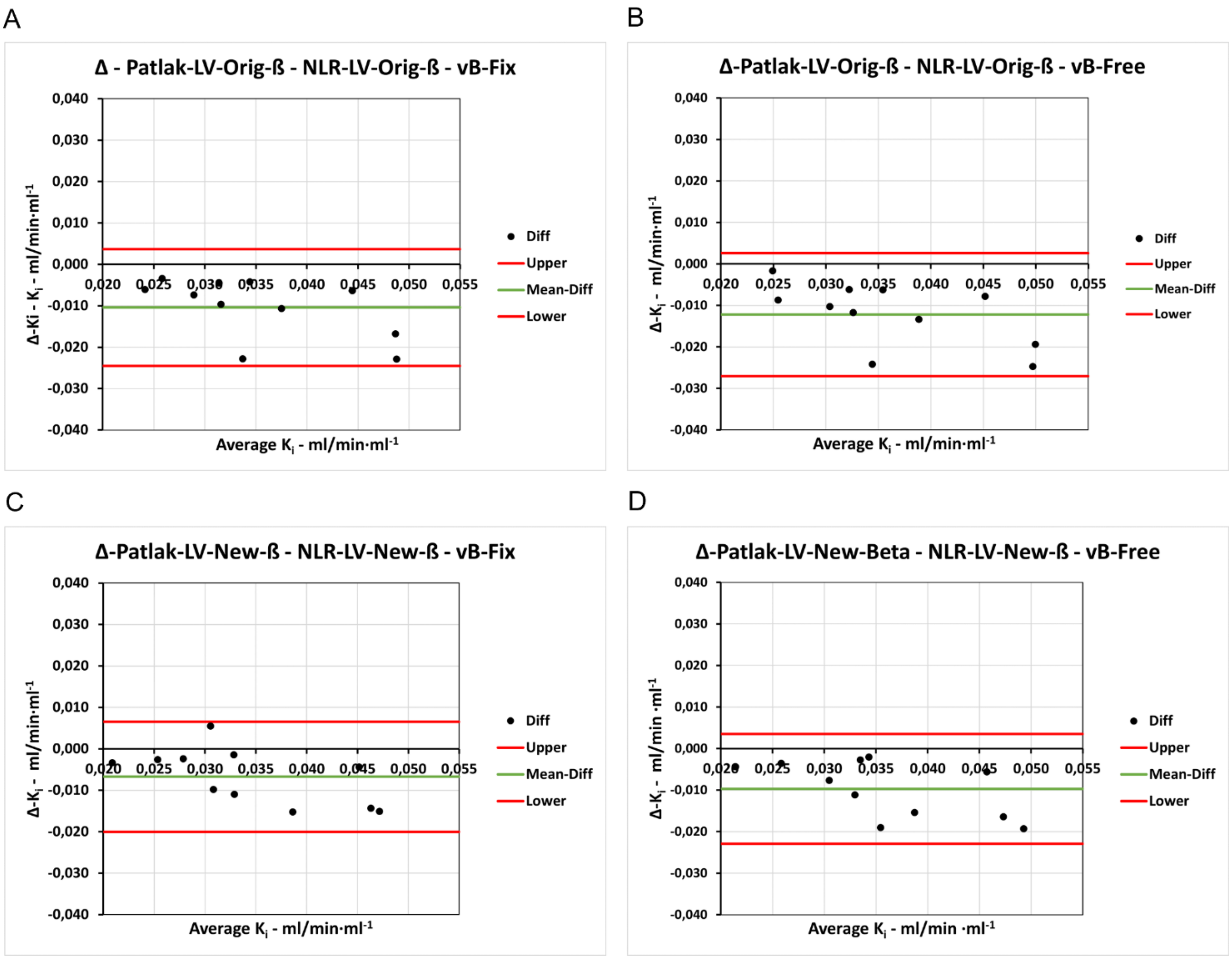

Figure 5 (E-H). Comparison of optimized NLR-Ki-values with the corresponding Patlak-values. E and F: LV-Fix-CF IDIFs with either fixed vB (left panel) or free-fitted vB (right panel). G and H: AO-Fix-ß IDIFs with either fixed vB (left panel) or free-fitted vB (right panel).

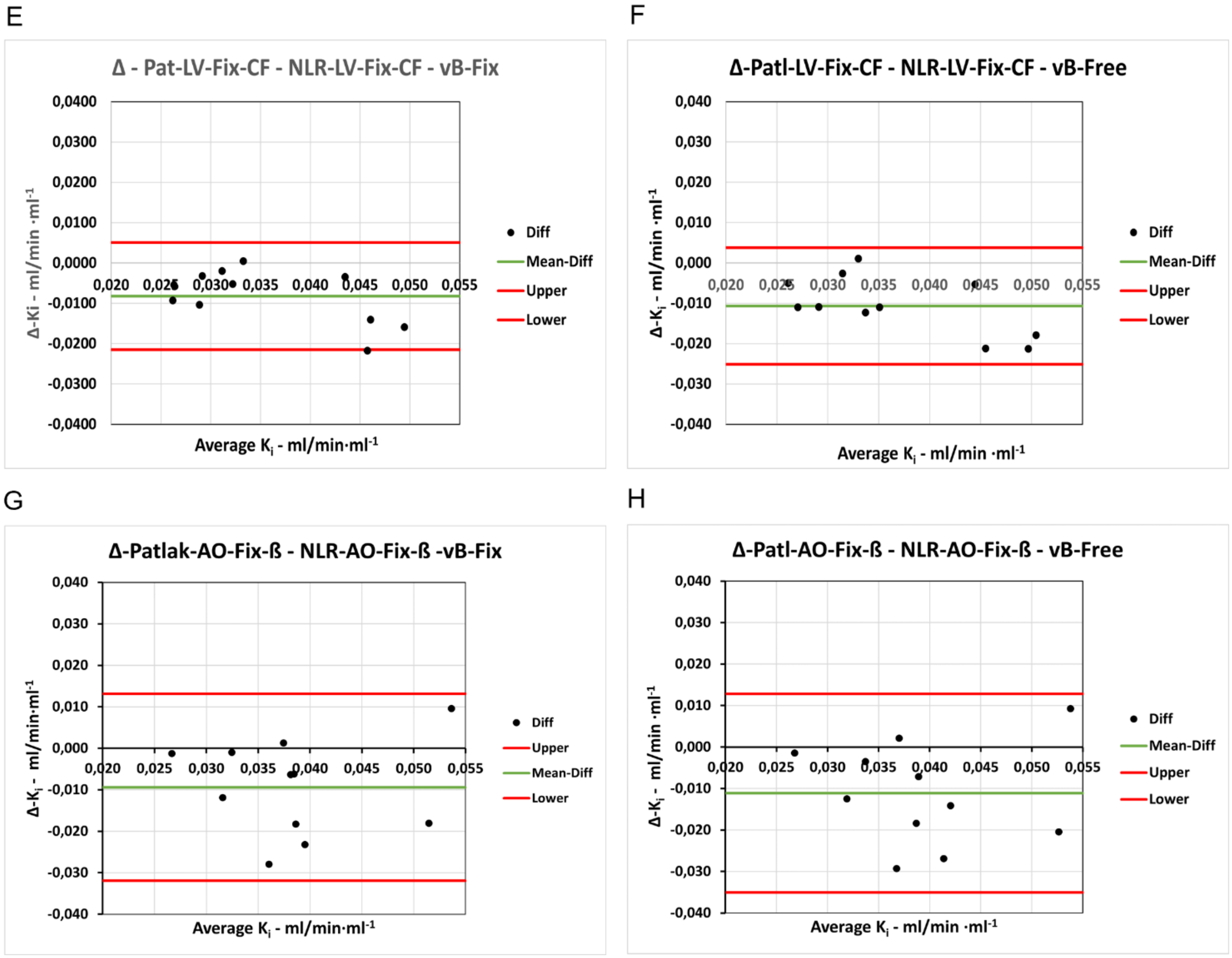

Table 6.A Bland-Altman quantitative analysis. Differences between the selected Patlak-IDIFs with their corresponding NLR-analysis with fixed vB. All Results in K_i ± SD ml/min·ml^{-1}. CL_{UL} and CL_{LL}: Upper and lower 95%-confidence limit. CI: Confidence interval.

LV-Orig-ß – Fix vB	Mean-Diff	SD	CL_{UL}	CL_{LL}	CI
**Patl-Basic**	-0.0104	0.0072	0.0037	-0.0245	0.0282
**Pat-PL-40-90-Log (Ki-60)**	-0.0106	0.0070	0.0031	-0.0243	0.0274
**Pat-PL-40-90-Exp (Ki-60)**	-0.0106	0.0070	0.0032	-0.0244	0.0276

LV-New-ß – Fix vB	Mean-Diff	SD	CL_{UL}	CL_{LL}	CI
**Patl-Basic**	-0.0067	0.0068	0.0065	-0.0200	0.0265
**Pat-PL-40-90-Log (Ki-60)**	-0.0065	0.0069	0.0071	-0.0202	0.0272
**Pat-PL-40-90-Exp (Ki-60)**	-0.0066	0.0069	0.0069	-0.0202	0.0271

LV-Fix-CF – Fix vB	Mean-Diff	SD	CL_{UL}	CL_{LL}	CI
**Patl-Basic**	-0.0082	0.0087	0.0051	-0.0215	0.0266
**Pat-PL-40-90-Log (Ki-60)**	-0.0083	0.0075	0.0064	-0.0230	0.0294
**Pat-PL-40-90-Exp (Ki-60)**	-0.0071	0.0084	0.0092	-0.0235	0.0327

AO-Fix-ß – Fix vB	Mean-Diff	SD	CL_{UL}	CL_{LL}	CI
**Patl-Basic**	-0.0094	0.0115	0.0132	-0.0319	0.0451
**Pat-PL-40-90-Log (Ki-60)**	-0.0109	0.0107	0.0100	-0.0319	0.0418
**Pat-PL-40-90-Exp (Ki-60)**	-0.0105	0.0109	0.0108	-0.0318	0.0427

Table 6.B Bland-Altman quantitative analysis. Differences between the selected Patlak-IDIFs with their corresponding NLR-analysis with free-fitted vB. All Results in $K_i \pm$ SD ml/min·ml^{-1}. CL_{UL} and CL_{LL}: Upper and lower 95%-confidence limit. CI: Confidence interval.

LV-Orig-ß – Free vB	Mean-Diff	SD	CL_{UL}	CL_{LL}	CI
**Patl-Basic**	-0.0122	0.0076	0.0026	-0.0270	0.0296
**Pat-PL-40-90-Log (Ki-60)**	-0.0124	0.0073	0.0019	-0.0266	0.0285
**Pat-PL-40-90-Exp (Ki-60)**	-0.0123	0.0074	0.0021	-0.0268	0.0289

LV-New-ß – Free vB	Mean-Diff	SD	CL_{UL}	CL_{LL}	CI
**Patl-Basic**	-0.0097	0.0067	0.0035	-0.0229	0.0264
**Pat-PL-40-90-Log (Ki-60)**	-0.0095	0.0069	0.0039	-0.0230	0.0269
**Pat-PL-40-90-Exp (Ki-60)**	-0.0096	0.0068	0.0038	-0.0230	0.0268

LV-Fix-CF – Fix vB	Mean-Diff	SD	CL_{UL}	CL_{LL}	CI
**Patl-Basic**	-0.0107	0.0074	0.0038	-0.0251	0.0289
**Pat-PL-40-90-Log (Ki-60)**	-0.0108	0.0083	0.0055	-0.0271	0.0326
**Pat-PL-40-90-Exp (Ki-60)**	-0.0096	0.0092	0.0083	-0.0276	0.0359

AO-Fix-ß – Free vB	Mean-Diff	SD	CL_{UL}	CL_{LL}	CI
**Patl-Basic**	-0.0111	0.0122	0.0128	-0.0350	0.0478
**Pat-PL-40-90-Log (Ki-60)**	-0.0127	0.0114	0.0096	-0.0349	0.0445
**Pat-PL-40-90-Exp (Ki-60)**	-0.0122	0.0116	0.0105	-0.0349	0.0454
